# Mesozoic lacewings from China provide phylogenetic insight into evolution of the Kalligrammatidae (Neuroptera)

**DOI:** 10.1186/1471-2148-14-126

**Published:** 2014-06-09

**Authors:** Qiang Yang, Yongjie Wang, Conrad C Labandeira, Chungkun Shih, Dong Ren

**Affiliations:** 1 College of Life Sciences, Capital Normal University, Beijing 100048, China; 2 Department of Paleobiology, National Museum of Natural History, Smithsonian Institution, Washington, DC 20013, USA; 3 Department of Entomology, University of Maryland, College Park, MD 20742, USA; 4 Geoscience Museum, Shijiazhuang University of Economics, Shijiazhuang 050031, China

**Keywords:** Jiulongshan formation, Yixian formation, Mouthparts, Wing eyespots, Phylogenetic analysis, Classification

## Abstract

**Background:**

The Kalligrammatidae are distinctive, large, conspicuous, lacewings found in Eurasia from the Middle Jurassic to mid Early Cretaceous. Because of incomplete and often inadequate fossil preservation, an absence of detailed morphology, unclear relationships, and unknown evolutionary trends, the Kalligrammatidae are poorly understood.

**Results:**

We describe three new subfamilies, four new genera, twelve new species and four unassigned species from the late Middle Jurassic Jiulongshan and mid Early Cretaceous Yixian Formations of China. These kalligrammatid taxa exhibit diverse morphological characters, such as mandibulate mouthparts in one major clade and siphonate mouthparts in the remaining four major clades, the presence or absence of a variety of distinctive wing markings such as stripes, wing spots and eyespots, as well as multiple major wing shapes. Based on phylogenetic analyses, the Kalligrammatidae are divided into five principal clades: Kalligrammatinae Handlirsch, 1906, Kallihemerobiinae Ren & Engel, 2008, Meioneurinae subfam. nov., Oregrammatinae subfam. nov. and Sophogrammatinae subfam. nov., each of which is accorded subfamily-level status. Our results show significant morphological and evolutionary differentiation of the Kalligrammatidae family during a 40 million-year-interval of the mid Mesozoic.

**Conclusion:**

A new phylogeny and classification of five subfamilies and their constituent genera is proposed for the Kalligrammatidae. These diverse, yet highly specialized taxa from northeastern China suggest that eastern Eurasia likely was an important diversification center for the Kalligrammatidae. Kalligrammatids possess an extraordinary morphological breadth and panoply of adaptations during the mid-Mesozoic that highlight our conclusion that their evolutionary biology is much more complex than heretofore realized.

## Background

The Kalligrammatidae are occasionally referred to as “butterflies of the Jurassic” [1] because of their large size and wingspans, presence of wings with patterned surfaces, including eyespots, and long maxillary palps. These features provide an appearance similar to some large, modern lepidopterans [1]. Kalligrammatids also have a mosaic of structural features displayed in other, various neuropterans. Their siphonate, long-proboscid mouthparts reported here for the first time, do not occur in modern Neuroptera, although the long, curved and lanciform ovipositor that occurs in *Oregramma illecebrosa* sp. nov. also is present among the modern pleasing lacewings of the Dilaridae [2]. Nevertheless, kalligrammatid lacewings, known only as compression fossils from the late Middle Jurassic (165 Ma) to mid Early Cretaceous (125 Ma), are an insect clade that exhibited a wide spectrum of diverse forms and appearances during the mid-Mesozoic, with 15 genera previously reported from Eurasia, including Western Europe, Central Asia, and Northeastern China [3-28] (Table 1).

**Table 1:** **Hierarchical classification of the 49 documented species of Kalligrammatidae**^**1**^

**Family Kalligrammatidae**	**Distribution**	**Geological age**
**Subfamily Kalligrammatinae Handlirsch, 1906**		
*Angarogramma*[28]		
*A. incertum*[28]	Ulan-Mayloulus, Russia	J_2_/Uda Fm.
*Kalligramma*[9]		
*K. brachyrhyncha* sp. nov.	Inner Mongolia, China	J_2_/Jiulongshan Fm.
*K. circularia* sp. nov.	Inner Mongolia, China	J_2_/Jiulongshan Fm.
*K. flexuosum*[15]	Karatau, Kazakhstan	J_3_/Karabastau Fm.
*K. haeckeli*[9]	Solnhofen, Germany	J_3_/Solnhofen Fm.
*K. jurarchegonium*[26]	Liaoning, China	J_2_/Haifanggou Fm.
*K. liaoningense*[21]	Beipiao City, China	K_1_/Yixian Fm.
*K. multinerve*[15]	Karatau, Kazakhstan	J_3_/Karabastau Fm.
*K. paradoxum*[27]	Inner Mongolia, China	J_2_/Jiulongshan Fm.
*K. roycrowsoni*[8]	Quarry Hill, England	K_1_/Wadhurst Clay Fm.
*K. sharovi*[15]	Karatau, Kazakhstan	J_3_/Karabastau Fm.
*K. turutanovae*[13]	Kazakhstan	J_3_/Karabastau Fm.
*Kalligramma sp.	Inner Mongolia, China	J_2_/Jiulongshan Fm.
*Kalligrammina*[16]		
*K. areolata*[16]	Karatau, Kazakhstan	J_3_/Karabastau Fm.
*Limnogramma*[20]		
*L. hani*[19]	Inner Mongolia, China	J_2_/Jiulongshan Fm.
*L. mira*[20]	Beipiao City, China	K_1_/Yixian Fm.
*L. mongolicum*[19]	Inner Mongolia, China	J_2_/Jiulongshan Fm.
*Sinokalligramma*[25]		
*S. jurassicum*[25]	Inner Mongolia, China	J_2_/Jiulongshan Fm.
**Subfamily Kallihemerobiinae Ren & Engel, 2008**		
*Affinigramma* gen. nov.		
*A. myrioneura* sp. nov.	Inner Mongolia, China	J_2_/Jiulongshan Fm.
*Apochrysogramma*[24]		
*A. rotundum*[24]	Inner Mongolia, China	J_2_/Jiulongshan Fm.
*Huiyingogramma*[27]		
*H. formosum*[27]	Inner Mongolia, China	J_2_/Jiulongshan Fm.
*Kalligrammula*[13]		
*K. atra*[17]	Mongolia	K_1_/Shine-Khuduk Fm.
*K. karatavica*[13]	Karatau, Kazakhstan	J_3_/Karabastau Fm.
*K. senckenbergiana*[5]	Solnhofen, Germany	J_3_/Solnhofen Fm.
*Kallihemerobius*[22]		
*K. aciedentatus* sp. nov.	Inner Mongolia, China	J_2_/Jiulongshan Fm.
*K. almacellus* sp. nov.	Inner Mongolia, China	J_2_/Jiulongshan Fm.
*K. feroculus* sp. nov.	Inner Mongolia, China	J_2_/Jiulongshan Fm.
*K. pleioneurus*[22]	Inner Mongolia, China	J_2_/Jiulongshan Fm.
*Lithogramma*[15]		
*L. oculatum*[15]	Karatau, Kazakhstan	J_3_/Karabastau Fm.
*Stelligramma* gen. nov.		
*S. allochroma* sp. nov.	Inner Mongolia, China	J_2_/Jiulongshan Fm.
*Kallihemerobiinae gen. indet.	Inner Mongolia, China	J_2_/Jiulongshan Fm.
**Subfamily Meioneurinae subfam. nov.**		
*Meioneurites*[4]		
*M. schlosseri*[4]	Solnhofen, Germany	J_3_/Solnhofen Fm.
*M. spectabilis*[11]	Karatau, Kazakhstan	J_3_/Karabastau Fm.
*M. villosus*[15]	Karatau, Kazakhstan	J_3_/Karabastau Fm.
**Subfamily Oregrammatinae subfam. nov.**		
*Abrigramma* gen. nov.		
*A. calophleba* sp. nov.	Pingquan, Hebei, China	K_1_/Yixian Fm.
*Ithigramma* gen. nov.		
*I. multinervia* sp. nov.	Inner Mongolia, China	K_1_/Yixian Fm.
*Ithigramma sp.	Inner Mongolia, China	K_1_/Yixian Fm.
*Oregramma*[20]		
*O. aureolusa* sp. nov.	Inner Mongolia, China	K_1_/Yixian Fm.
*O. gloriosa*[20]	Beipiao City, China	K_1_/Yixian Fm.
*O. illecebrosa* sp. nov.	Beipiao City, China	K_1_/Yixian Fm.
*Oregramma sp.	Inner Mongolia, China	K_1_/Yixian Fm.
**Subfamily Sophogrammatinae subfam. nov.**		
*Protokalligramma*[24]		
*P. bifasciatum*[24]	Inner Mongolia, China	J_2_/Jiulongshan Fm.
*Sophogramma*[21]		
*S. eucallum*[21]	Beipiao City, China	K_1_/Yixian Fm.
*S. lii*[23]	Beipiao City, China	K_1_/Yixian Fm.
*S. papilionacea*[21]	Beipiao City, China	K_1_/Yixian Fm.
*S. pingquanica* sp. nov.	Pingquan, Hebei, China	K_1_/Yixian Fm.
*S. plecophlebia*[21]	Beipiao City, China	K_1_/Yixian Fm.
*Sophogramma sp. [12]	Baissa, Russia	K_1_/Zaza Fm.
Uncertain subfamily		
*Palparites*[4]		
*P. deichmuelleri*[4]	Solnhofen, Germany	J_3_/Solnhofen Fm.

^1^*Abbreviations*: *Designates an uncertain species; J_2_, Middle Jurassic, J_3_, Late Jurassic, K_1_, Early Cretaceous.

Although the Kalligrammatidae are readily identified by their distinctive appearance, there still are no definitive, conventional diagnoses available. Some genera and species erected by early systematists and assigned to the Kalligrammatidae were poorly described and based solely on fragmentary wings. There are conflicting relationships among genera and species within the family. For example, the monotypic genus *Palparites* was erected by [4] based on a poorly preserved specimen and an obfuscating description. Lambkin [6] reassigned the specimen to the Kalligrammatidae, and mentioned that the specimen shares general characters with other kalligrammatids, such as a large body size and a dense network of crossveins. However, the systematic position of this genus within the Kalligrammatidae remains uncertain. Because of the rarity and quality of specimens, it has been difficult to conduct a comprehensive review of this family.

Recently, a large number of well-preserved kalligrammatid specimens have been described from the Mesozoic of China [19-27], augmenting our knowledge of the family. Here, we describe sixteen additional species, including three unassigned to a species and one unassigned to a genus from northeastern China ranging in age from late Middle Jurassic (Jiulongshan Formation, 165 Ma) to the mid Early Cretaceous (Yixian Formation, 125 Ma). We redefine the family based on synapomorphic characters, and present a dichotomous key and classification of the genera. We conducted a phylogenetic analysis, and the results partition the Kalligrammatidae into five subfamilies: Kalligrammatinae Handlirsch, 1906 [4], Kallihemerobiinae Ren and Engel [29], Meioneurinae subfam. nov., Oregrammatinae subfam. nov. and Sophogrammatinae subfam. nov. Our goal is to explore the relationships of various extinct kalligrammatid taxa, using a broad sample of well-described material. Our study emphasizes the late Middle Jurassic of Daohugou, Inner Mongolia Autonomous Region in northeastern China (Jiulongshan Fm.), the mid Late Jurassic of Karatau, from southern Kazakhstan (Karabastau Fm.), and the mid Early Cretaceous of Liaoning and Hebei Provinces in northeastern China (Yixian Fm.). The Kalligrammatidae exhibit an elevated speciosity and broad morphological diversity during the mid-Mesozoic, suggesting these large insects were a significant ecologic component within the ecosystems they inhabited.

## Results

### Descriptions of specimens

The authors for the new taxa established below shall be Yang, Wang, Labandeira, Shih and Ren.

Wing nomenclature and abbreviations used in the text are as follows: 1A–3A, anal veins; C, costa; Cu, cubitus; CuA, anterior cubitus; CuP, posterior cubitus; M, media; MA, anterior branch of media; MP, posterior branch of media; ORB, oblique radial branch of anterior radial trace = "radial sector"; pt, pterostigma; R, radius; R1, first branch of radius; Rs, radial sector; Sc, subcosta; Vr, humeral recurrent vein [30-32].

Order **Neuroptera** Linnaeus, 1758

Family **Kalligrammatidae** Handlirsch, 1906

Type genus: ***Kalligramma*** Walther, 1904

#### Redescription

Large insects (body length more than 50 mm); densely setose throughout the body and wings. Antennae filiform, usually not exceeding the length of forewing. Mouthparts mandibulate, or more commonly siphonate and forming a prominent proboscis from conjoined maxillary galeae. Forewing exceptionally large and broad, more than 50 mm long, approximately triangular or oviform in outline; usually bearing distinct, prominent eyespots positioned in the centers of the wing. Costal region expanded, costal veinlets dichotomously branched distally (with the exception of the Oregrammatinae), interlinked by numerous smaller veinlets. Sc and R1 fused distally; r1-rs crossveins numerous; the basal stem of Rs and R1 usually fused, with the exception of *Affinigramma* gen. nov., *Apochrysogramma* and *Kallihemerobius* where the separation of Rs from R1 is distal from wing base. MA typically originating from Rs as in most neuropterans, except for *Affinigramma* gen. nov. and *Kallihemerobius*, with MA diverging from R1; ORB diverging from R1, forming a few pectinate branches, present in some genera of subfamily Kallihemerobiinae; MP extensively branched, MP_2_ with many distal pectinate branches, consisting of an expansive, triangular region, regarded as an autapomorphy of Kalligrammatidae (except for *Sophogramma*). Cu forked near wing base; CuA and CuP forming complex secondary branches. Anal region broad; 1A parallel to the posterior margin, with complexly bifurcating branches. Hind wing generally triangular; costal veinlets typically simple or with few distal bifurcations.

#### Remarks

The extinct family Kalligrammatidae is characterized by remarkably large body size, extremely broad wing shape, unusually dense venation and complex secondary branches of longitudinal veins, which allow separation from other families by their branching pattern. In general, specimens of Kalligrammatidae are poorly preserved with overlapping, incomplete or fragmentary wings. Some species historically were poorly described, resulting in an incomplete understanding of the morphological breadth within the family. The exact definition of the family is imprecise because of the allocation of taxa to varied groups and poor descriptions of many species. One example is the genus *Angarogramma*, erected by Ponomarenko in 1984, although the venation in the published drawing is overly simplified to provide useful information [28], the specimen still could be attributed to the Kalligrammatidae from the distinctive wing shape. But its generic status is doubtful, as it lacks valid diagnostic characters. For this reason, we propose the above diagnosis of the Kalligrammatidae for unambiguous differentiation of this family from other families of Neuroptera.

Subfamily **Kallihemerobiinae** Ren & Engel, 2008

**Type genus.***Kallihemerobius* Ren & Oswald, 2002.

**Included genera.** Type genus and *Affinigramma* gen. nov., *Apochrysogramma* Yang, Makarkin & Ren, 2011, *Huiyingogramma* Liu et al., 2013, *Kalligrammula* Martynova, 1947, *Lithogramma* Panfilov, 1968, and *Stelligramma* gen. nov.

#### Diagnosis

No convincing characters uniquely define the Kallihemerobiinae. However, the parsimony analysis below indicates that the deep MA bifurcation indicates monophyly of the Kallihemerobiinae. However, establishment of robust characters for the Kallihemerobiinae monophyly requires further testing with additional specimens and data.

Genus ***Affinigramma*** gen. nov.

(urn:lsid:zoobank.org:act:4221DD6B-A562-4CC0-BBD8-D89F292D8815)

**Type species**. *Affinigramma myrioneura* sp. nov.

#### Etymology

The generic name is derived from the Latin word *affinis*, meaning “similar” or “related”, and the Greek *gramma*, meaning “lined” or “written”, referring to wings that resemble a manuscript, also a common suffix for kalligrammatid genera. Gender feminine.

#### Diagnosis

Siphonate proboscis present. Humeral recurrent veins (Vr) absent; all costal veinlets sinuate and forked. Rs with at least 6 pectinately forked branches. Only one, the ORB, between Rs and MA. MA originating from R1, initially dichotomously forked, then pectinately forked and continuing to vein end. CuA single; CuP pectinately forked. 1A pectinately forked.

#### Remarks

*Affinigramma myrioneura* gen. et sp. nov. is placed in the subfamily Kallihemerobiinae by having the following characters: 1), the longitudinal veins bear marginal branching along the wing margin; 2), crossveins are not present in the area of marginal branching; 3), the forewing has forked c-sc crossveins; and 4), the Sc terminates in the R1 near the wing apex. The new genus differs from *Kallihemerobius*[22] in the following characters: 1), all costal veinlets are sinuate and bear a sparsely branched, humeral recurrent crossvein; 2), the Rs is branched from the R1 only, but not with multiple “Rs” veins as in *Kallihemerobius*; 3), only one pectinately forked ORB is present; 4), the MA branches separately from the R1; 5), the CuA is single; and 6), all anal veins are pectinately forked.

**Figure 1:**
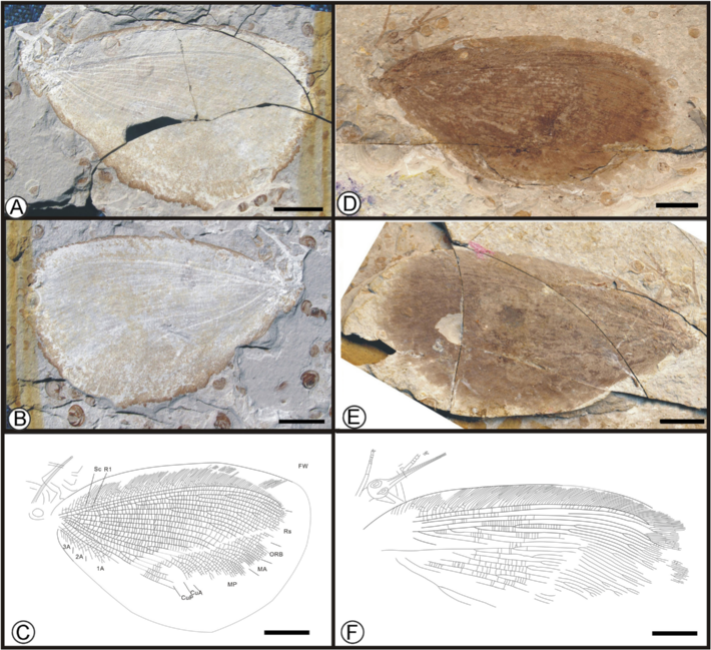
**Images and camera lucida drawings of *Affinigramma myrioneura *sp. nov. A, B, C, Holotype CNU-NEU-NN2009-006P/C; D, E, F, Paratype CNU-NEU-NN2009-007P/C.** Scale bars, 10 mm.

***Affinigramma myrioneura*** sp. nov. (Figure 1)

(urn:lsid:zoobank.org:act:344C60A3-F25E-4FB6-A2E8-32B17A2653C7)

**Diagnosis.** As for the genus, by monotypy.

#### Etymology

The specific epithet of *myrioneura* is derived from the Greek words *myrio*, meaning “countless” or “numberless”, and *neura*, meaning a “sinew” or “nerve”, referring to the complex venation of this species.

#### Holotype

Specimen (part and counterpart) with partial body and one forewing; specimen CNU-NEU-NN2009-006P/C (Figure 1A, B). Paratype with four, superimposed wings; well-preserved head and leg segments (part and counterpart); specimen CNU-NEU-NN2009-007P/C (Figure 1D-F). The sex is unknown for both specimens.

#### Type locality and horizon

Jiulongshan Formation, Middle Jurassic; Daohugou Village, Shantou Township, Ningcheng County, Inner Mongolia, China.

#### Measurements

Holotype, CNU-NEU-NN2009-006P/C (Figure 1A, B): forewing ca. 62 mm long, 40 mm wide. Paratype, CNU-NEU-NN2009-007P/C (Figure 1D, E): forewing ca. 68 mm long, 35 mm wide as preserved.

#### Description

Large insects, body partly preserved in lateral view (Figure 1C); forewing nearly complete. Head elongate; antennae not preserved. Siphonate proboscis present. Legs partially preserved; structure unclear. Dense crossveins throughout the wings. Forewing broadly ovoidal in shape, with rounded apex. Humeral recurrent veins (Vr) absent, all costal veinlets sinuate and forked. R1 simple, parallel to Sc for a long distance, fused apically, and then curved posteriorly to enter margin before wing apex; Rs with ca. 6 primary branches, all pectinately forked. Only one ORB between Rs and MA, with ca. three pectinate branches. MA originating from R1, initially dichotomously forked, then pectinately forked distally; MP sinuate, with at least 6 branches, the first simple, the others pectinately forked. CuA single; CuP pectinately forked, with more than 4 branches. Between CuA and CuP an intercalary vein originates from CuP and ends at CuP prior to branching. 1A with many pectinately forked branches; 2A and 3A simple, all pectinately forked. Wing eyespot obvious on forewing.

Genus ***Kallihemerobius*** Ren & Oswald, 2002

**Type species.***Kallihemerobius pleioneurus* Ren & Oswald, 2002

**Included species.** Type species and *Kallihemerobius aciedentatus* sp. nov., *Kallihemerobius almacellus* sp. nov., and *Kallihemerobius feroculus* sp. nov.

**Figure 2:**
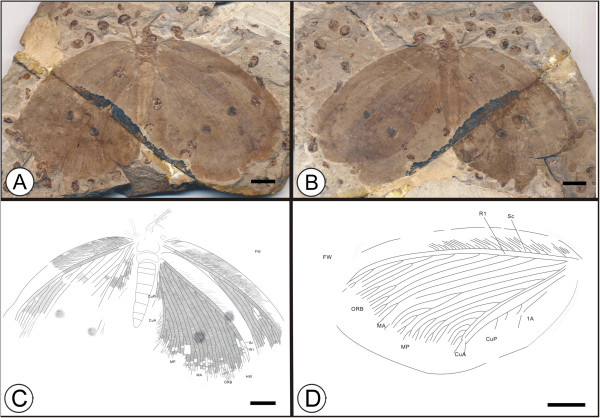
**Images and camera lucida drawings of *Kallihemerobius aciedentatus *sp. nov. CNU-NEU-NN2010-008P/C; A, B, Holotype; C, Outline of holotype; D, Outline of forewing.** Scale bars, 10 mm.

***Kallihemerobius aciedentatus*** sp. nov. (Figure 2)

(urn:lsid:zoobank.org:act:3554140B-1919-49D8-BC31-E3248D5A8E10)

#### Diagnosis

Mouthparts siphonate. Forewing with 8 ORB between Rs and MA; MP with 9 pectinate branches, the second pectinately forked. Hind wing with 8 ORB, all dichotomously forked; MA with 3 pectinate branches.

#### Etymology

The specific epithet, *aciedentatus*, comes from the Latin *acies*, meaning “sharp” or “edge”, and *dentatus*, meaning “tooth-like” or “having teeth”.

#### Holotype

Specimen (part and counterpart) with partial body and four wings, specimen CNU-NEU-NN2010-008P/C (Figure 2A, B). Sex unknown.

#### Type locality and horizon

Jiulongshan Formation, Middle Jurassic, Daohugou Village, Shantou Township, Ningcheng County, Inner Mongolia, China.

#### Measurements

Holotype, CNU-NEU-NN2010-008P/C (Figure 2A, B): forewing 67 mm long and 32 mm wide as preserved; hind wing ca. 61 mm long, 38 mm wide as preserved.

#### Description

Head elongate; antennae filiform, one preserving nine articles (Figure 2C); compound eye large, occupying ca. half of head length; malar space elongate. Proboscis complete, galeae disassociated. Legs partly preserved. Abdomen covered by hind wing, poorly preserved.

Forewing oviform (Figure 2D) Dense crossveins throughout the wing. Humeral recurrent veins (Vr) absent, all costal veinlets sinuate and deeply forked. R1 simple, parallel to Sc for a long distance and fused apically; 8 ORB between Rs and MA, all dichotomously forked. MA originating from R1, simple; MP sinuate, with 9 pectinate branches. CuP with several pectinate branches. Wing spot obvious on forewing, consisting of one black, circular splotch.

Hind wing oviform. Humeral recurrent veins (Vr) absent; all costal veinlets sinuate and deeply dichotomously forked. R1 simple, parallel to Sc for a substantial distance and fused apically, then curved posteriorly toward the wing margin; Rs bifurcate in the preserved part, with 8 ORB all dichotomously forked. MA originating from R1, forked at vein midsection, with 3 pectinate branches; MP sinuate, with at least 7 outwardly directed pectinate branches, the first simple, the others dichotomously forked. CuA with at least 10 pectinate branches; CuP not clear. A not preserved.

#### Remarks

*Kallihemerobius aciedentatus* sp. nov. is one of the best-preserved specimens examined, with four wings extended and detailed body structures present. It differs from other species in the following characters: 1), the siphonate proboscis and palpi are long and narrow; 2), the forewing Rs has eight ORB, all dichotomously forked; 3), the forewing MP vein bears nine pectinate branches, the second pectinately forked; 4), the hind wing Rs vein has eight ORB, all dichotomously forked; and 5), the hind wing MA vein subtends three pectinate branches.

**Figure 3:**
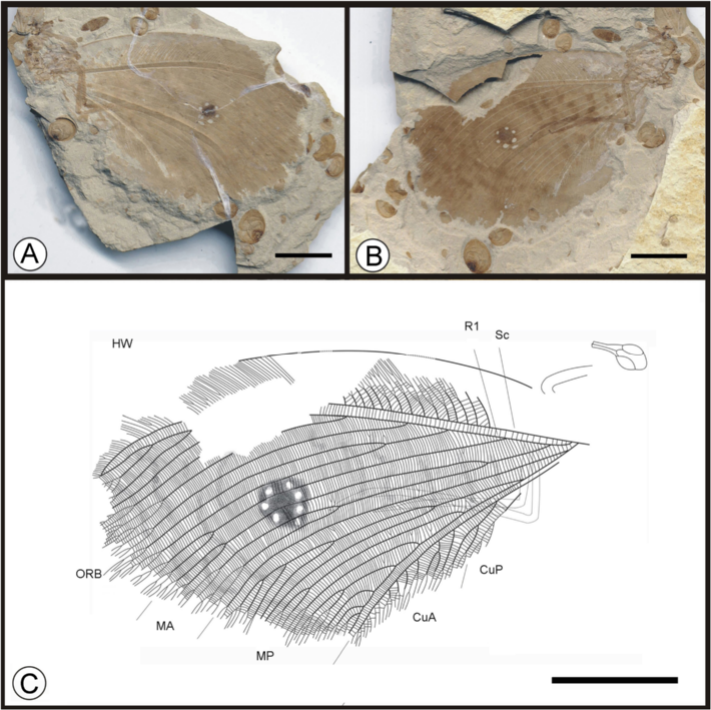
**Images and camera lucida drawing of *Kallihemerobius almacellus *sp. nov. CNU-NEU-NN2009-050P/C. A**, **B**, Holotype; **C**, Outline of holotype. Scale bars, 10 mm.

***Kallihemerobius almacellus*** sp. nov. (Figure 3)

(urn:lsid:zoobank.org:act:3628F2E7-7898-46E6-97 F2-D07AD9E00CD5)

#### Diagnosis

Head with substantial malar space; siphonate proboscis present. Hind wing lacking humeral recurrent veins (Vr). Rs bearing at least 3 pectinate branches; 7 ORBs between Rs and MA. MP sinuate, comprising 9 branches, the first simple, the second with 4 pectinately forked branches, the others dichotomously forked. CuA with at least 13 pectinate branches. Wing eyespot conspicuous.

#### Etymology

The specific epithet, *almacellus*, is derived from the Latin *alma*, meaning “nourishing” or “kind”, and *cella*, meaning “a cell” or “a small enclosure”, referring to the abundant cells within the wings.

#### Holotype

Specimen (part and counterpart) with partial body and one hind wing; specimen CNU-NEU-NN2009-050P/C (Figure 3A, B). Sex unknown.

#### Type locality and horizon

Jiulongshan Formation, Middle Jurassic, Daohugou Village, Shantou Township, Ningcheng County, Inner Mongolia, China.

#### Measurements

Holotype, CNU-NEU-NN2009-050P/C (Figure 3A, B): hind wing ca. 46 mm long, 30 mm wide as preserved.

#### Description

Head elongate, small (Figure 3C); antennae not preserved; compound eyes large, occupying ca. half of head length; malar space significant. Siphonate proboscis, with overlapping maxillary palps. Leg elements variably preserved. Hind wing well preserved. Wing eyespot obvious. Hind wing oviform. Humeral recurrent veins (Vr) absent; all costal veinlets sinuate and deeply dichotomously forked. R1 simple, parallel to Sc for a long distance and fused apically, then curved posteriorly toward wing margin; Rs comprised of at least 3 pectinate branches; 7 ORBs between Rs and MA; ORB_2_ forked much earlier than others. MA originating from R1, forked near wing base, with two dichotomously forked branches; MP sinuate, with 9 branches, the first simple, the second with 4 pectinately forked branches, the others dichotomously forked. CuA with at least 13 pectinate branches; CuP not clear. A veins not preserved.

#### Remarks

*Kallihemerobius almacellus* sp. nov. differs from other species in the following characters: 1), the costal region is broader; 2), seven ORBs are present; 3), the second branch of MP is pectinately forked; and 4), the CuA subtends at least thirteen pectinate branches.

**Figure 4:**
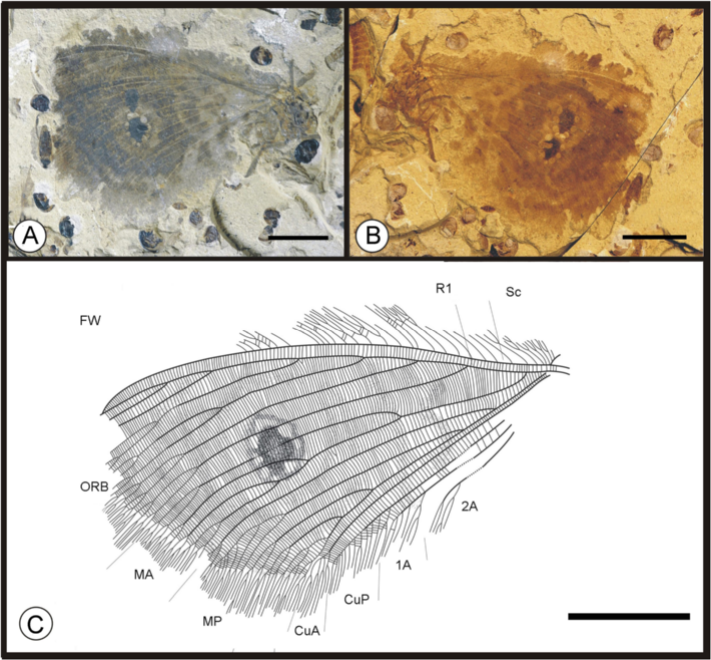
**Images and camera lucida drawing of *Kallihemerobius feroculus *sp. nov. CNU-NEU-NN2010-013P/C. A**, **B**, Holotype; **C**, Outline of holotype. Scale bars, 10 mm.

***Kallihemerobius feroculus*** sp. nov. (Figure 4)

(urn:lsid:zoobank.org:act:1D58ECE7-DE1F-46FB-B869-1E3B0455FF79)

#### Diagnosis

Forewing bearing humeral recurrent veins (Vr) and 5 ORBs; ORB_3_ forked earlier than others. MA forked nearly 1/3 the distance from the wing base; MP sinuate, the second branch with ca. 6 pectinate branches, the others dichotomously forked. CuA and CuP pectinately forked. 1A and 2A pectinately forked. Wing eyespot prominent; a central, dark pigmented area surrounded by a ring.

#### Etymology

The specific epithet, *feroculus*, is from the diminutive Latin Word, *feroculus*, meaning “fierce” or “savage”.

#### Holotype

A specimen (part and counterpart) with partial body and one forewing; specimen CNU-NEU-NN2010-013P/C (Figure 4A, B). Sex unknown.

#### Type locality and horizon

Jiulongshan Formation, Middle Jurassic, Daohugou Village, Shantou Township, Ningcheng County, Inner Mongolia, China.

#### Measurements

Holotype, CNU-NEU-NN2010-013P/C (Figure 4A, B): forewing ca. 37 mm long, 26 mm wide as preserved.

#### Description

A well-preserved forewing with a missing border. Body partially preserved. Head and mouthparts preserved ventrally (Figure 4C).

Forewing oviform. Humeral recurrent veins (Vr) present, all costal veinlets sinuate and deeply forked. R1 simple, parallel to Sc for a significant distance and fused apically; Rs simple in preserved part; 5 ORBs, ORB_3_ with 6 primary pectinate branches, all dichotomously forked; Rs_3_ forked earlier than others. MA originating from R1, forked nearly 1/3 the distance from wing base, with 3 pectinate branches, each dichotomously forked; MP sinuate, with 5 pectinate branches, the second with ca. 6 pectinate branches, the others dichotomously forked. CuA pectinately forked near wing margin; CuP with ca. 10 pectinate branches. 1A and 2A parallel to CuP and pectinately forked; 3A absent or not preserved. Wing eyespot obvious, enveloped by a circular ring.

#### Remarks

*Kallihemerobius feroculus* sp. nov. differs from other species in the following characters: 1), only five ORBs are present; 2), the MA is forked considerably earlier; 3), the MP subtends five pectinate branches; 4), the CuA is pectinately forked near the wing margin; 5), the CuP has ca. ten pectinate branches; and 6), the 1A and 2A veins are pectinately forked.

Genus ***Stelligramma*** gen. nov.

(urn:lsid:zoobank.org:act:D4606681-1755-422 F-BDE9-A94F4C97B43F)

**Type species.***Stelligramma allochroma* sp. nov.

#### Etymology

The generic name is derived from the Latin *stella*, meaning “star” or “astral”, and the Greek *gramma*, meaning “lined” or “written”, referring to wings resembling a manuscript; also a common suffix for kalligrammatid genera. Gender feminine.

#### Diagnosis

Forewing without eyespot markings. Humeral recurrent veins (Vr) evident. Most costal veinlets curved, deeply forked near wing margin, bearing one to three interlinked veinlets. Rs with pectinate branches, all bifurcate. MA forked at vein midsection. Cu forked into two branches at wing base; CuA pectinately forked terminally and CuP dichotomously forked at midvein. 1A deeply dichotomously forked; 2A pectinately forked, each branch dichotomously forked; 3A simple.

#### Remarks

The genus *Stelligramma* gen. nov. has a common kalligrammatid-like appearance, and was assigned to the subfamily Kallihemerobiinae. The kallihemerobiine assemblage is complex for its varied wing structure, including the absence of the ORB, and narrow costal region. Based on our phylogenetic analysis, the monophyly of subfamily Kallihemerobiinae is supported by a single character: proximal forking of the 1A vein. *Stelligramma* gen. nov. and *Kalligrammula* constitute the stem-group of Kallihemerobiinae, representing the earliest divergence of the subfamily. This divergence implies that absence of the ORB among *Stelligramma* gen. nov. and *Kalligrammula* is likely an ancestral feature.

**Figure 5:**
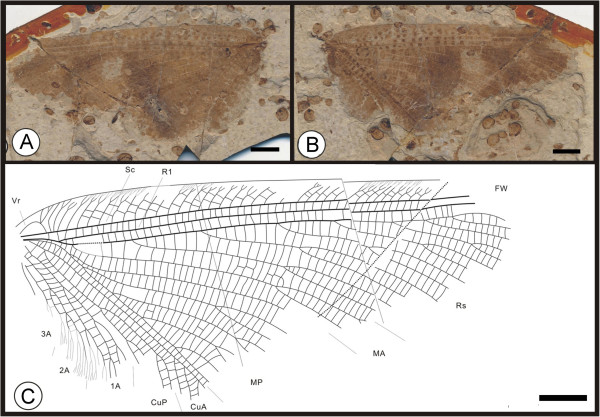
**Images and camera lucida drawing of *Stelligramma allochroma *sp. nov. CNU-NEU-NN2010-012P/C. A**, **B**, Holotype; **C**, Outline of holotype. Scale bars, 10 mm.

***Stelligramma allochroma*** sp. nov. (Figure 5)

(urn:lsid:zoobank.org:act:910C978D-F8C3-4D26-B9DD-BC627FDB8A35)

**Diagnosis.** As for the genus, by monotypy.

#### Etymology

The epithet of *allochroma* is derived from the Greek words, *allos*, meaning “other” or “different”, and *chroma*, meaning “color” or “hue”.

#### Holotype

A well-preserved forewing (part and counterpart); specimen CNU-NEU-NN2010-012P/C (Figure 5A, B). Sex unknown.

#### Type locality and horizon

Jiulongshan Formation, Middle Jurassic, Daohugou Village, Shantou Township, Ningcheng County, Inner Mongolia, China.

#### Measurements

Holotype, CNU-NEU-NN2010-012P/C (Figure 5A,B): forewing ca. 90 mm long, 44 mm wide.

#### Description

One partially preserved forewing (Figure 5C). Axial body not preserved. Forewing approximately obtuse-triangular. Eyespot or spot absent. Humeral recurrent veins (Vr) evident. Most costal veinlets curved and deeply forked at wing margin, between which are one or two interlinked veinlets. R1 parallel to Sc where preserved. Rs with more than 7 original branches, all bifurcate. MA forked at vein midsection; MP forked near wing base, with at least 5 pectinate branches, the first simple, the others dichotomously forked. Cu forked into two branches at wing base; CuA parallel with MP and pectinately forked at end; CuP dichotomously forked at vein midsection. 1A forked earlier than CuP, deeply dichotomously forked; 2A with ca. 3 pectinate branches, each dichotomously forked; 3A simple. Wing spot obvious on forewing, consisting of one black, circular splotch.

**Figure 6:**
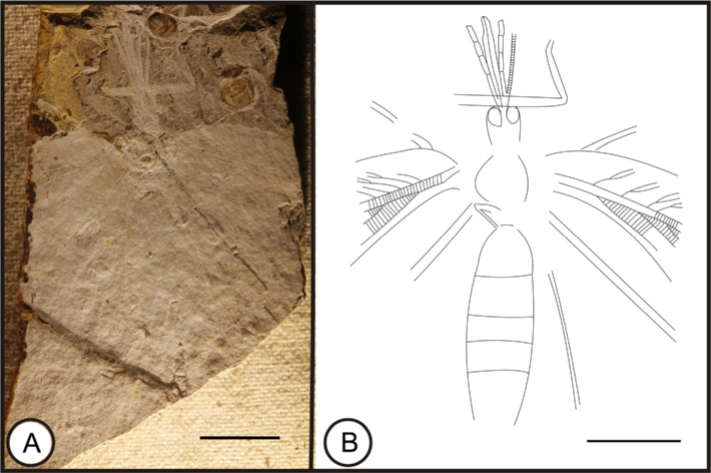
**Image and camera lucida drawings of Kallihemerobiinae gen. et sp. indet. CNU-NEU-NN2009-033. A**, Habitus; **B**, Outline of habitus. Scale bars, 10 mm.

**Kallihemerobiinae** gen. et sp. indet. (Figure 6)

#### Material

A mostly poorly preserved specimen with partial body and wing impression; specimen CNU-NEU-NN2009-033 (Figure 6A). Sex unknown.

#### Type locality and horizon

Jiulongshan Formation, Middle Jurassic, Daohugou Village, Shantou Township, Ningcheng County, Inner Mongolia, China.

#### Description

Body preserved in ventral view (Figure 6B). Head better preserved; compound eyes hemispheroidal, modest; proboscis present, with maxillary palps; antenna filiform, only left antenna (partly) preserved. Legs incompletely preserved. Dense crossveins throughout the wings. Forewing costal region broad; all costal veinlets sinuate, deeply forked; other structures indistinct. Forewing spot or eyespot absent.

#### Remarks

The presence of a siphonate proboscis and an ORB suggests that this specimen unequivocally belongs to the Kallihemerobiinae. However, other parts of the specimen are so poorly preserved that they lacks diagnostic information to determine the specimens’ generic status. We assign this specimen as gen. et sp. indet. to the Kallihemerobiinae.

Subfamily **Kalligrammatinae** Handlirsch, 1906

**Type genus:***Kalligramma* Walther, 1904

#### Included genera

Type genus and *Angarogramma* Ponomarenko, 1984; *Kalligrammina* Panfilov, 1980; *Limnogramma* Ren, 2003; and *Sinokalligramma* Zhang, 2003.

#### Diagnosis

Apomorphic characters defining the Kalligrammatinae are: 1), distal pectinate branching in the anterior media (MA) vein; 2), complex branching along the distal course of the anterior cubitus (CuA) vein; and 3), complex branching along the distal course of the posterior cubitus (CuP) vein.

#### Remarks

The subfamily consists of five genera typical of the Kalligrammatidae. The Kalligrammatinae is distinguished from the other subfamilies by the following characters: 1), a broad triangular MP region versus parallel MP branches among the Sophogrammatinae; 2), absence of an ORB versus presence of an ORB among Kallihemerobiinae; and 3), a complex costal cross-venation versus simple costal cross-venation among Meioneurinae and Oregrammatinae. Although the establishment of the Kalligrammatinae is based on a combination of the above apomorphic characters, this taxon currently lacks a synapomorphy.

Genus ***Kalligramma*** Walther, 1904

Type species ***Kalligramma haeckeli*** Walther, 1904

#### Included species

Type species and *Kalligramma brachyrhyncha* sp. nov., *Kalligramma circularia* sp. nov., *Kalligramma flexuosum* Panfilov, 1968; *Kalligramma jurarchegonium* J. Zhang & H. Zhang, 2003, *Kalligramma liaoningense* Ren & Guo, 1996, *Kalligramma multinerve* Panfilov, 1968, *Kalligramma roycrowsoni* Jarzembowski, 2001, *Kalligramma sharovi* Panfilov, 1968, *Kalligramma turutanovae* Martynova, 1947. *Kalligramma brachyrhyncha* sp. nov.; *Kalligramma circularia* sp. nov.

**Figure 7:**
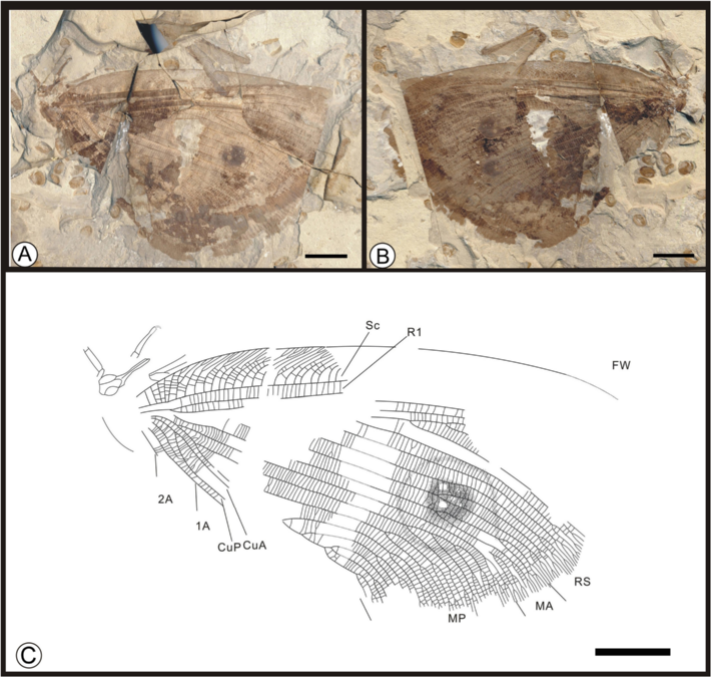
**Images and camera lucida drawing of *Kalligramma brachyrhyncha *sp. nov. CNU-NEU-NN2009-030P/C. A**, **B**, Holotype; **C**, Outline of holotype. Scale bars, 10 mm.

***Kalligramma brachyrhyncha*** sp. nov. (Figure 7)

(urn:lsid:zoobank.org:act:620F475B-7CFB-4A32-AD77-DC6CBEF3D94C)

#### Diagnosis

Most costal veinlets curved and forking multichotomously; interlinked veinlets between costal veinlets abundant. Rs with more than 6 primary branches; MP forked near wing base, with 6 pectinate branches, the first forked later than the second. Cu divided into CuA and CuP near base; one intercalary vein between Cu and 1A.

#### Etymology

The specific epithet of *brachyrhyncha* is derived from the Greek words of “*brachy*”, meaning “short” or “abbreviated”, and *rhynchos*, for “beak” or “mouth”.

#### Holotype

A partially preserved specimen (part and counterpart) with four overlapping wings and incomplete body; specimen CNU-NEU-NN2009-030P/C (Figure 7A,B). Sex unknown.

#### Type locality and horizon

Jiulongshan Formation, Middle Jurassic, Daohugou Village, Shantou Township, Ningcheng County, Inner Mongolia, China.

#### Measurements

Holotype, CNU-NEU-NN2009-030P/C (Figure 7A,B): siphonate proboscis present; forewing approximately 73 mm long, 42 mm wide; hind wing ca. 71 mm long, 42 mm wide.

#### Description

Body preserved in lateral view (Figure 7C). Antennae not preserved. Siphonate proboscis shorter than the maxillary palps. Thorax and abdomen poorly preserved. Forewings and hind wings overlapping; most of forewing discernible. Forewing approximately obtuse-triangular. Most costal veinlets curved and forked. Rs with more than 6 primary branches; forked near wing apex. MA with 2 pectinate branches at posterior margin; MP forked close to wing base, with 6 pectinate branches, the first forked later than the second. At the preserved wing base, Cu divided into CuA and CuP, and one intercalary vein between Cu and 1A. Anal region indistinct; 1A simple, as preserved. Wing eyespot obvious on forewing, consisting of light colored ovate ocules within and surrounding the central pigmented area, in turn enveloped by a circular, darkly pigmented ring at some distance. Hind wing broad, nearly acute-triangular; preservation fragmentary, only the overall outline and apical region identifiable. In the preserved part, most costal veinlets curved and forked. Sc and R1 fused apically and then curved posteriorly to enter margin before wing apex, Rs exceeding 5 branches; each sequentially forked.

#### Remarks

*Kalligramma brachyrhyncha* sp. nov. is attributable to the genus *Kalligramma* based on the above-mentioned characters. *Kalligramma brachyrhyncha* sp. nov. is similar to *K. turutanovae*[13] in forewing structure, although *K. turutanovae* is preserved without a hind wing. In the new species, the Rs has more than six primary branches, whereas *K. turutanovae* has more than eight. In the new species the MP is forked near the wing base, with six pectinate branches, the first forked later than the second, whereas in *K. turutanovae* the MP has three visible branches, and the first is forked earlier than the second. In the new species, the Cu is divided into a CuA and a CuP near the wing base, and one intercalary vein occurs between the Cu and 1A; by contrast, in *K. turutanovae* both the CuA and CuP are pectinately forked.

*Kalligramma brachyrhyncha* sp. nov. resembles *K. multinerve*[15] in forewing features. The new species has most costal veinlets curved, forking multichotomously, and interlinked with all regional veinlets, whereas *K. multinerve* has costal veinlets forked twice, lacking crossveins posteriorly, and Sc and R1 are not fused in the preserved material. In the new species, the MP has six pectinate branches, but in *K. multinerve* the MP has three visible branches. For the new species the Cu is divided into a CuA and a CuP near the wing base, and one intercalary vein occurs between the Cu and 1A, but in *K. multinerve* the CuA is simple.

**Figure 8:**
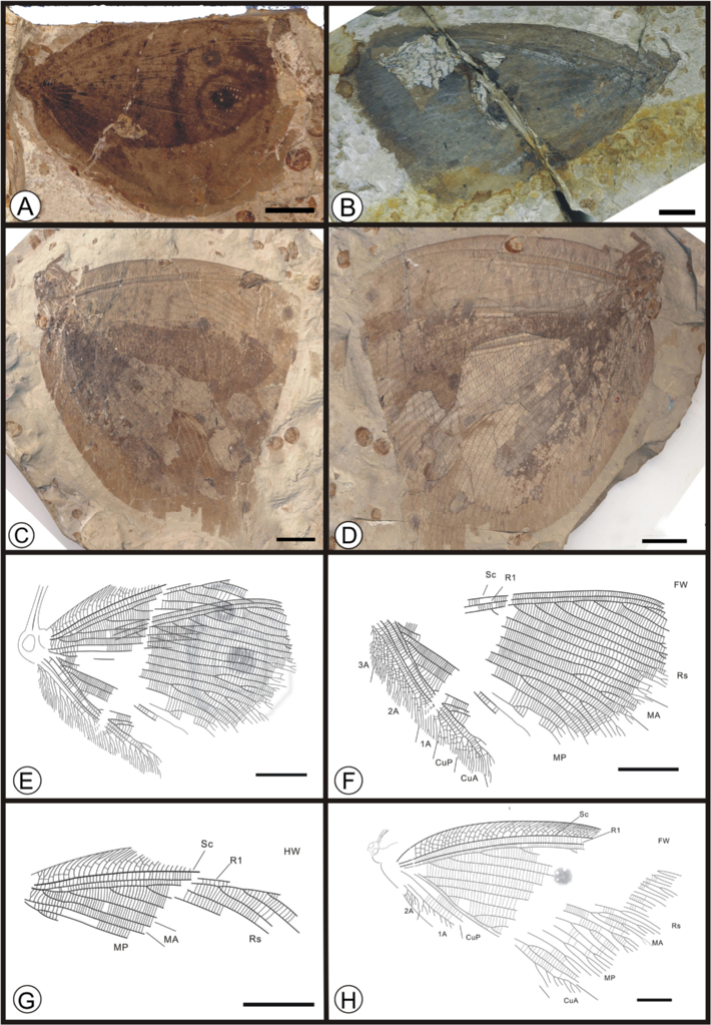
**Images and camera lucida drawings of *Kalligramma circularia *sp. nov. A, Holotype CNU-NEU-NN2010-003; B, Paratype CNU-NEU-NN2010-011; C, D, Paratype CNU-NEU-NN2010-015P/C; E, Outline of holotype; F, Forewing of holotype; G, Hind wing of holotype; H, Outline of paratype CNU-NEU-NN2010-011.** Scale bars, 10 mm.

***Kalligramma circularia*** sp. nov. (Figure 8)

(urn:lsid:zoobank.org:act:01A5669B-7C4C-4 F20-99B0-BDE6B98A639B)

#### Diagnosis

Forewing costal margin with an obvious structural camber. Most costal veinlets forked, with few interlinked veinlets. Rs with 12 original branches; MP with more than 9 pectinate branches, the second pectinately forked, with 6 branches. Both CuA and CuP pectinately forked at the wing margin. 1A pectinately forked.

#### Etymology

The specific epithet originates from the Latin “*circularis*”, meaning “circular”, “round” or an “orb”, emphasizing the large, round wing eyespots of this species.

#### Holotype

A partially preserved specimen with four overlapping wings and an incomplete body; specimen CNU-NEU-NN2010-003 (Figure 8A). Paratype specimens CNU-NEU-NN2010-015P/C (Figure 8C,D) and CNU-NEU-NN2010-011 (Figure 8B). The sex is unknown for all specimens.

#### Type locality and horizon

Jiulongshan Formation, Middle Jurassic, Daohugou Village, Shantou Township, Ningcheng County, Inner Mongolia, China.

#### Measurements

Holotype, CNU-NEU-NN2010-003 (Figure 8A): forewing ca. 65 mm long, 54 mm wide as preserved. Paratypes, CNU-NEU-NN2010-015P/C (Figure 8C, D): proboscis preserved. CNU-NEU-NN2010-011 (Figure 8B): proboscis present, forewing ca. 87 mm long, 49 mm wide.

#### Description

Body preserved in lateral view (Figure 8E). Head partially preserved, structures ambiguous; mouthparts not preserved. Compound eyes hemispheroidal and prominent; antennae not preserved. Thorax and abdomen poorly preserved. Femora and tibiae of legs preserved. Forewings and hind wings overlapping, most forewing features discernible; wings with abundant crossveins throughout.

Forewing of holotype approximately obtuse-triangular (Figure 8F). Most costal veinlets curved and forked; few interlinked veinlets. Sc and R1 parallel, fused apically and subsequently curved posteriorly toward the margin. Rs with 12 primary branches, forked at wing apex. MA exceeding 3 pectinate branches; stem of MP, CuA and CuP not preserved. Anal region indistinct; 1A with many pectinate branches. Wing eyespot obvious. Hind wing preservation fragmentary for the holotype (Figure 8G). Hind wing of paratype CNU-NEU-NN2010-011 with relatively narrow costal region (Figure 8H); most costal veinlets curved and forked, with several interlinked veinlets. R1 parallel to Sc; Rs with more than 7 pectinate branches. MA originating from a barely discernible R1.

#### Remarks

*Kalligramma circularia* sp. nov. is similar to *K. multinerve*[15] in forewing features, the latter of which is preserved without a hind wing. It is different from *K. multinerve* in the following characters: 1), most of the new species’ costal veinlets are forked, with few interlinked veinlets; 2), the Rs has 12 primary branches; 3), the MP subtends more than nine pectinate branches, the second pectinately forked, with six branches; and 4), both the CuA and CuP are pectinately forked at the wing margin.

**Figure 9:**
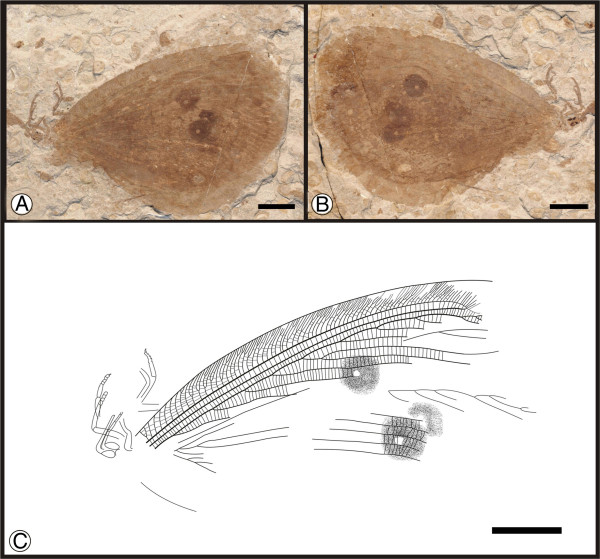
**Images and camera lucida drawing of *Kalligramma *sp. CNU-NEU-NN2010-010P. A, B, Habitus; C, Outline of habitus.** Scale bars, 10 mm.

***Kalligramma*** sp. (Figure 9)

#### Material

A partially preserved specimen with four overlapping wings and incomplete body (part and counterpart); specimen CNU-NEU-NN2010-010P/C (Figure 9A,B). The sex is unknown.

#### Type locality and horizon

Jiulongshan Formation, Middle Jurassic, Daohugou Village, Shantou Township, Ningcheng County, Inner Mongolia, China.

#### Measurements

CNU-NEU-NN2010-010P/C (Figure 9A,B): forewing 64 mm long, 37 mm wide as preserved.

#### Description

Body preserved in lateral view (Figure 9C). Compound eyes large; antennae fragmentary. Proboscis incomplete. Thorax and abdomen not preserved; portions of forelegs and midlegs preserved. Four wings overlapping; most venation difficult to discern, but apparently crossveins occur throughout the wings.

Forewing approximately obtuse-triangular. Most costal veinlets curved and forked, with dense interlinked veinlets. R1 parallel to Sc for a considerable distance, then fused apically and curved posteriorly toward wing margin. Rs with ca. 10 primary pectinate branches. Wing eyespot conspicuous on forewing.

#### Remarks

The specimen *Kalligramma* sp. is deemed an indeterminate species, as overlapping wings obscure most of the determinative vein characters.

Subfamily **Oregrammatinae** subfam. nov.

(urn:lsid:zoobank.org:act:6707AFBB-C7AB-4217-879 F-89D57DE947A0)

**Type genus.***Oregramma* Ren, 2003

#### Included genera

Type genus, *Abrigramma* gen. nov., and *Ithigramma* gen*.* nov.

#### Diagnosis

Apomorphic characters defining the Oregrammatinae are: 1), the costal region is strongly constricted toward the wing apex; 2), the second branch of the posterior media vein (MP2) is basally arched; and 3), a broadening of the cubitus region occurs along the midwing interval.

Genus ***Abrigramma*** gen. nov.

(urn:lsid:zoobank.org:act:3172C76C-AF70-468D-92 DC-A99B4F220BFD)

**Type species.***Abrigramma calophleba* sp. nov.

#### Diagnosis

Humeral recurrent veins (Vr) absent. Forewing Rs with at least 9 primary branches, all single. MA pectinately forked, having a false origin from MP. 2A with ca. 13 pectinately forked branches. Hind wing Rs almost originating from R1 at 1/4 of the distance from its base, branching at ca. apical third of wing length, forming at least 2 primary branches. MP first branch forked nearly at base of wing, third branch terminates at the wing’s posterior border, before wing apex. 2A with at least 9 pectinate branches; 3A short, with 3 pectinate, dichotomous branches.

#### Etymology

The generic name is derived from the French *abri*, a “shelter” or “haven”, a modification of the Latin, *apricum*, for “an open place”, referring to the spreading wings of this species; and the Greek, *gramma,* meaning “lined” or “written”, referring to wings provided with a manuscript-like appearance, also a common suffix of kalligrammatid genera. The gender is feminine.

#### Remarks

This new genus and new species, *Abrigramma calophleba* gen. et sp. nov., is placed in the Kalligrammatidae based on the following characters: 1), the forewing is remarkably large, with a costal area of moderate width; 2), the R1 is unbranched; 3), the Rs has numerous pectinate branches; and 4) the MP is extensively branched and provided with several main branches anteriorly and numerous crossveins over the entire wing. The hind wing is noticeably large, with its costal area demonstrably larger than representatives of related families. In addition, the R1 is unbranched, the Rs has few pectinate branches, the MA is forked shallowly along the posterior part of the wing, the MP is pectinately forked, and abundant crossveins occur throughout the wing.

**Figure 10:**
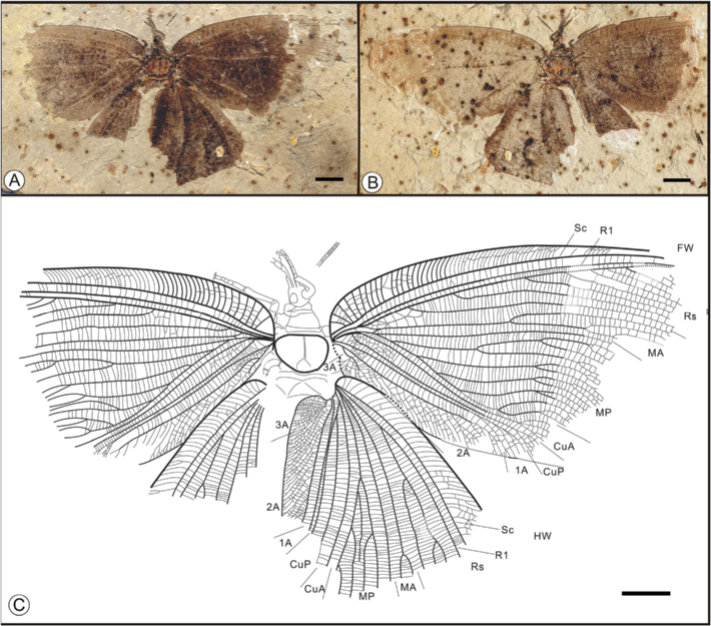
**Images and camera lucida drawing of *Abrigramma calophleba *sp. nov. CNU-NEU- HP2009-001P. A**, **B**, Holotype; **C**, Outline of holotype. Scale bars, 10 mm.

***Abrigramma calophleba*** sp. nov. (Figure 10)

(urn:lsid:zoobank.org:act:3FF1C294-4C34-48FA-B957-A08B2C47055E)

#### Diagnosis

As for the genus, by monotypy.

#### Etymology

The specific epithet, *calophleba*, is derived from the Greek, *kalos*, for “beautiful”, and *phleba*, for “vein” of a leaf or insect wing.

#### Holotype

A well-preserved specimen (part and counterpart) with most of the forewings preserved; hind wings are present and the axial body is incomplete; specimen CNU-NEU-HP2009-001P/C (Figure 10A,B). The sex is unknown.

#### Type locality and horizon

Yixian Formation, mid Early Cretaceous, Pingquan County, Chengde City, Hebei Province, China.

#### Measurements

Holotype, CNU-NEU-HP2009-001P/C (Figure 10A, B): forewing the preserved portion of length 73 mm and width 44 mm, hind wing 45 mm long, 41 mm wide as preserved.

#### Description

A moderately large insect; body partially preserved in dorsal view (Figure 10C). Compound eyes large; antennae filiform. Mouthparts with long, robust proboscis and palpi. Prothorax short, trapeziform; mesothorax pyriform, wider than prothorax. Left foreleg almost complete, with 5 tarsal segments. Setae densely investing body and wings.

Forewing approximately triangular-obtuse, apical angle and outer margin partly missing. Wing eyespot faint. Humeral recurrent veins (Vr) absent; all costal veinlets sinuate, only several forked. R1 parallel to Sc almost to wing margin; Rs traceable almost to base of R1, with at least 9 primary branches, all single. MA pectinately forked, with 3 branches, having a false origin from MP; MP sinuate, with 6 pectinate branches. CuA forked trifurcately near wing margin; CuP branched near wing margin, with 2 dichotomously pectinate branches. 1A with 3 branches; 2A with ca. 13 pectinate branches, occupying most of the posterior wing margin; 3A short and unbranched. Wing spot obvious, consisting of one black, circular splotch.

Hind wing short and broad, almost triangular-acute; posterior area absent. All costal veinlets sinuate. R1 unbranched, parallel to Sc for a long distance; origin of Rs slightly distant from wing base. MA unbranched, dichotomously forked near wing margin; MP pectinately branching, with minimally 5 branches; first branch almost at wing base, third branch terminates at wing’s hind margin, before wing apex. CuA single; CuP originating from CuA, dichotomously forked. 1A unbranched; 2A with at least 9 pectinate branches; 3A short, with 3 pectinate, dichotomous branches.

Genus ***Ithigramma*** gen. nov.

(urn:lsid:zoobank.org:act:2DF7B863-BB52-46E4-950E-56D592AB7F77)

**Type species.***Ithigramma multinervia* sp. nov.

**Included species**. Type species and *Ithigramma* sp.

#### Etymology

The generic name is derived from the Greek, *ithys*, meaning “straight”, for the rectilinear course of the wing venation of this species; and the Greek, *gramma,* meaning “lined” or “written”, referring to wings provided with a manuscript-like appearance, also a common suffix of kalligrammatid genera. The gender is feminine.

#### Diagnosis

Large insects; densely setose or scaled throughout the body and wings. Antennae filiform, short. Mouthparts with elongate palpi, exceeding the head length. Wings with indistinct pterostigma, wing eyespot present.

#### Remarks

*Ithigramma* gen. nov. is different from other genera by the following features: 1), the mouthparts are significantly elongate, with palps longer than the head length; and 2), a dense vestiture of setae and scales covers the body and wings. The setae and scales on the wings are frequently so dense that veins are obscured.

**Figure 11:**
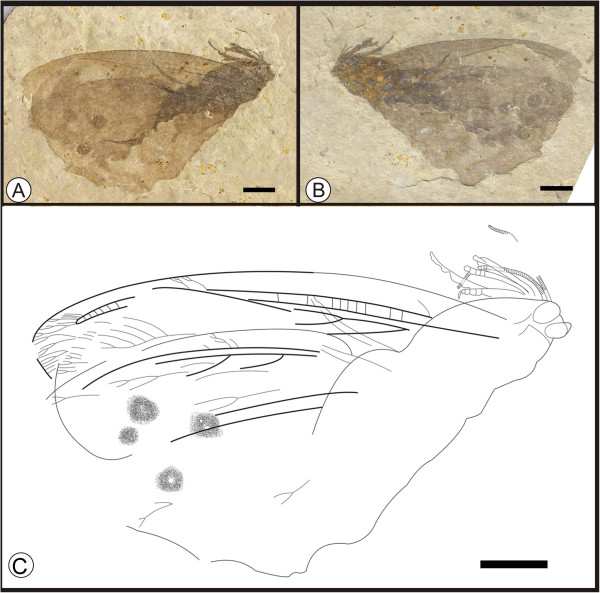
**Images and camera lucida drawing of *Ithigramma multinervia *sp. nov. CNU-NEU-NN2009-034P. A, B,** Holotype; **C**, Outline of holotype. Scale bars, 10 mm.

***Ithigramma multinervia*** sp. nov. (Figure 11)

(urn:lsid:zoobank.org:act:B98C978B-8A12-4B35-AD3D-9C20D692F076)

#### Diagnosis

Body robust, densely setose throughout, including the wings, obscuring the venation. Forewings and hind wings each with an eyespot. Forewing costal region narrowed; Sc and R1 fused apically and then curved posteriorly to enter wing margin. Dense crossveins occurring throughout the wings.

#### Etymology

The specific epithet, *multinervia*, is derived from the Latin *multus*, meaning “many”, and *nervus*, meaning “vein”.

#### Holotype

A poorly preserved specimen (part and counterpart), the structure of both the body and venation is quite indistinct; specimen CNU-NEU-NN2009-034P/C (Figure 11A, B). The sex is unknown.

#### Type locality and horizon

Yixian Formation, Early Cretaceous, Liutiaogou Village, Ningcheng County, Inner Mongolia, China.

#### Measurements

Holotype, CNU-NEU-NN2009-034P/C (Figure 11A, B): antennae 16 mm long as preserved; hind wing ca. 71 mm long, 34 mm wide.

#### Description

Large insects, body partially preserved in lateral view (Figure 11C). Entire body, including wings, densely setose throughout. Body robust; cephalic, thoracic and abdominal structures unclear. Compound eyes poorly defined; antennae filiform. Maxillary palps elongate, longer than head length. Forewings and hind wings overlapping, eyespots visible; venation indistinct, few veins discernible, the margin of each wing unclear. Dense crossveins occurring throughout the wings. Pterostigma unclear; wing eyespot on forewing.

Forewing approximately obtuse-triangular. Costal region narrow; Sc and R1 fused apically, curved posteriorly to enter margin; Rs bearing several pectinate branches.

**Figure 12:**
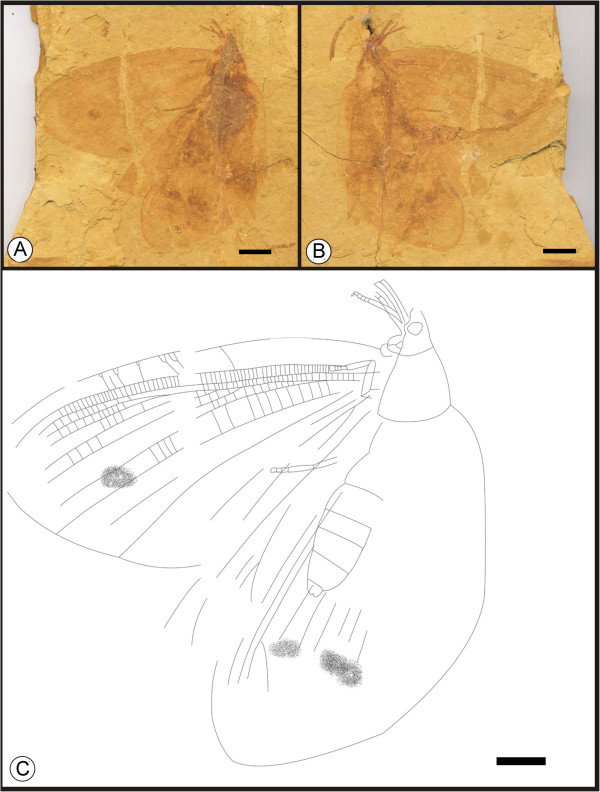
**Images and camera lucida drawing of *Ithigramma *sp. CNU-NEU-NN2010-016P. A**, **B**, Habitus; **C**, Outline of habitus. Scale bars, 10 mm.

***Ithigramma*** sp. (Figure 12)

#### Material

A poorly preserved specimen (part and counterpart); body structure and venation indistinct; specimen CNU-NEU-NN2010-016P/C (Figure 12A, B). Sex female.

#### Type locality and horizon

Yixian Formation, Early Cretaceous, Liutiaogou Village, Ningcheng County, Inner Mongolia, China.

#### Measurements

CNU-NEU-NN2010-016P/C (Figure 12A, B): forewing ca. 56 mm long, 29 mm wide as preserved.

#### Description

Large insects, robust body partially preserved in lateral view (Figure 12C); dense setae and scales throughout the body and overlapping wings. Antennae and compound eyes not identifiable; palpi elongate, longer than head length. Head and thorax structure unclear; abdomen with 5 discernible segments. Legs partly preserved. Venation indistinct, only several main veins recognizable. Dense crossveins occurring throughout the wings.

Forewing approximately obtuse-triangular, the apex smooth. Costal region not broad. Rs with several pectinate branches. Status of other veins not clear. Pterostigma faint; eyespot present on forewing.

#### Remarks

The veins of *Ithigramma* sp. are sparser than those of *Ithigramma multinervia* sp. nov. Because many characters are not clearly preserved, we cannot attribute this specimen to a species.

Genus ***Oregramma*** Ren, 2003

**Type species.***Oregramma gloriosa* Ren, 2003

#### Included species

Type species and *Oregramma aureolusa* sp. nov., *Oregramma illecebrosa* sp. nov., and *Oregramma* sp.

**Figure 13:**
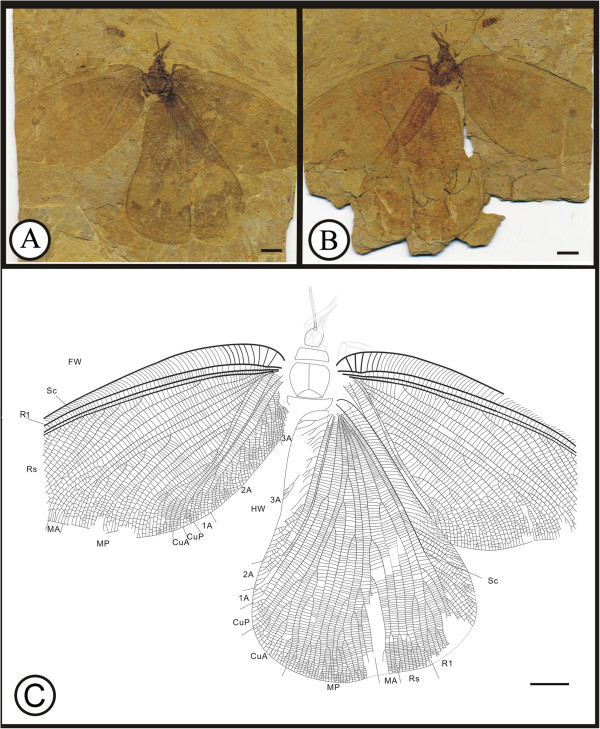
**Images and camera lucida drawing of *Oregramma aureolosa *sp. nov. CNU-NEU- NN2009-032P. A**, **B**, Holotype; **C**, Outline of holotype. Scale bars, 10 mm.

***Oregramma aureolusa*** sp. nov. (Figure 13)

(urn:lsid:zoobank.org:act:6046A8CC-4598-45D9-93AF-FB764358BBC7)

#### Diagnosis

Compound eyes large; palpi elongate. Forewing lacking humeral recurrent veins (Vr). MA pectinately forked; MP sinuate, coursing in an arc, with 8 pectinate branches. CuA and CuP branched near wing margin. 1A dichotomously forked near terminus; 2A pectinately forked. Forewing with eyespots; pterostigma indistinct. Hind wing pyriform in gross shape; all costal veinlets arched with some crossveins forking near posterior wing margin. R1 simple, with three upturned pectinate branches near terminus. MA dichotomously forked along middle section of wing.

#### Etymology

The specific epithet of *aureolusa* is from the Latin word “*aureolus*” meaning “made of gold”, or figuratively “golden” or “gorgeous”.

#### Holotype

A well-preserved specimen (part and counterpart) with most of the forewings present, one complete hind wing and an incomplete axial body; specimen CNU-NEU-NN2009-032P/C (Figure 13A, B). Sex probably male.

#### Type locality and horizon

Yixian Formation, Early Cretaceous, Liutiaogou Village, Ningcheng County, Inner Mongolia, China.

#### Measurements

Holotype, CNU-NEU-NN2009-032P/C (Figure 13A,B): antennae ca. 10.5 mm long; forewing 74.5 mm long, 41.5 mm wide as preserved. Preserved hind wing with length 69.0 mm, width 57.0 mm.

#### Description

Large insect, body partially preserved in dorsal view (Figure 13C); dense setae and scales covering body and wings. Antennae filiform, with 37 articles. Compound eyes large; proboscis present and palpi elongate. Prothorax short, trapeziform; mesothorax pyriform, wider than prothorax. Fragmentary leg segments preserved. Dense crossveins along all wings.

Forewing approximately obtuse-triangular, apical parts missing. Humeral recurrent veins (Vr) absent. All costal veinlets arched but not forked. R1 parallel to Sc in preserved part; Rs with at least 10 primary branches, all single in the central region. MA pectinately forked, with 3 branches; MP sinuate, forming an arc with 8 pectinate branches. CuA and CuP branched near wing margin. 1A dichotomously forked near terminus; 2A with ca. 13 pectinately forked branches; 3A with ca. three simple branches. Eyespot present, distinctive.

Hind wing pyriform in shape. All costal veinlets arched with some forked crossveins entering the apical area. R1 simple, parallel to Sc for a considerable distance, with three upturned pectinate branches terminally; Rs with three branches, the first branching ca. the apical third of wing length. MA dichotomously forked at vein midsection; MP pectinately branching, with 5 branches, first branch nearly at wing base. CuA forked at wing base, the first branch at wing midsection; the second with three pectinate branches at its base; CuP single. 1A simple; 2A with ca. 13 pectinate branches; 3A faint.

#### Remarks

*Oregramma aureolusa* sp. nov. is attributed to the genus *Oregramma* based on the above-mentioned characters. Also, it is distinguished from the only known forewing of this genus, *Oregramma gloriosa*[20], by having the following characters: 1), the MA is pectinately forked, with 3 branches; 2), the MP has 8 pectinate branches; 3), the Cu is divided into CuA and CuP near the wing base; 4), the 1A is dichotomously forked near its terminus; and 5), the 2A subtends ca. 13 pectinately forked branches.

**Figure 14:**
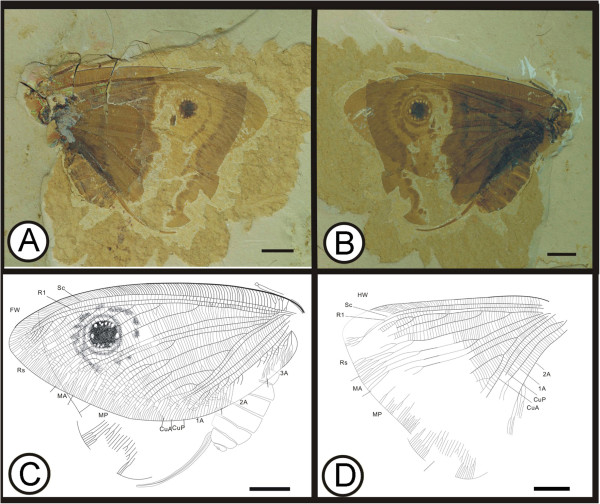
**Images and camera lucida drawings of *Oregramma illecebrosa *sp. nov. CNU-NEU-LB2009-031C. A**, **B**, Holotype; **C**, Outline of forewing with body; **D**, Outline of hind wing. Scale bars, 10 mm.

***Oregramma illecebrosa*** sp. nov. (Figure 14)

(urn:lsid:zoobank.org:act:24362312-4249-4EFF-9010-0ADEF2A33FBF)

#### Diagnosis

Siphonate proboscis covered with setae. Ovipositor distinctive; curved and robust. Forewing with prominent eyespots. Most costal veinlets arcuate, only several at the beginning and end are forked. MA with 3 sinuate and pectinate branches at posterior wing margin; MP forked near the wing base, with 6 pectinate branches. CuA and CuP simple, with occasional branching. 1A with 3 pectinate branches; 2A with ca. 8 pectinate branches; 3A with 3 dichotomous branches. Hind wing with Rs of ca. 5 branches, each sequentially forked. MA forked earlier than all branches of Rs, with two dichotomously forked branches; MP pectinately forked. 2A with many pectinate branches.

#### Etymology

The specific epithet of *illecebrosa* is derived from the Latin, “*illecebrosus*”, meaning “attractive” or “charming”.

#### Holotype

A well-preserved specimen (part and counterpart) with a nearly complete forewing, a fragmentary hind wing covered by forewing and incomplete body, specimen CNU-NEU-LB2009-031P/C (Figure 14A, B). Female.

#### Type locality and horizon

Yixian Formation, Early Cretaceous, Huangbanjigou, Beipiao City, Liaoning Province, China.

#### Measurements

Holotype, CNU-NEU-LB2009-031P/C (Figure 14A, B): ovipositor ca. 25 mm long; forewing 75 mm long, ca. 35 mm wide; hind wing ca. 65 mm long, 53 mm.

#### Description

Body preserved in lateral view (Figure 14C). Head incompletely preserved; antennae faint; incomplete. Maxillary palps mostly missing, some disarticulated articles present; proboscis setaceous. Thorax indistinct; mostly covered by setae. Abdomen well preserved, covered by a setal vestiture; 7 segments discernible and subterminal ovipositor prominent and robust, curved, with two pairs of valves present.

Forewing approximately obtuse-triangular, with obvious eyespot (Figure 14C). Most costal veinlets curved, several beginning and ending crossveins forked. Sc and R1 fused apically and then curved posteriorly to enter margin before the wing apex. Rs with 11 original branches, forked immediately prior to wing apex. MA with 3 sinuate and pectinate branches toward posterior border of wing; MP forked near wing base, with 6 pectinate branches, the first forked later than the second. CuA and CuP simple, with limited branching. 1A parallel to CuP for a significant distance with 3 pectinate branches; 2A parallel to 1A with ca. 8 pectinate branches; 3A with 3 dichotomous branches. Conspicuous wing eyespot on the forewing.

Hind wing broad, nearly acute-triangular (Figure 14D). Wing margin not evident, most of the hind wing covered by forewing. Most costal veinlets arrayed vertically, some near the apical region curved and forked. Anal veins overlapping body; margins unclear. Sc and R1 fused apically, then curved posteriorly to enter margin before wing apex; Rs with ca. 5 branches, each sequentially forked. MA forked earlier than all branches of Rs, with two dichotomously forked branches; MP with more than 4 pectinately forked branches. CuA parallel to CuP in the preserved part. 1A indistinct; 2A partly preserved, with many pectinate branches; 3A absent.

#### Remarks

*Oregramma illecebrosa* sp. nov. is probably one of the best preserved of kalligrammatid specimens. This species is assigned to the genus *Oregramma* based on the above characters. *O. illecebrosa* is considered a distinct species based on the following characters: 1), *O. illecebrosa* has a 1A pectinately forked whereas in *O. aureolusa* 1A is dichotomous near its terminus; and 2), *O. illecebrosa* has a 2A with ca. 8 pectinate branches, whereas in *O. aureolusa* 2A has ca. 13 pectinate branches.

**Figure 15:**
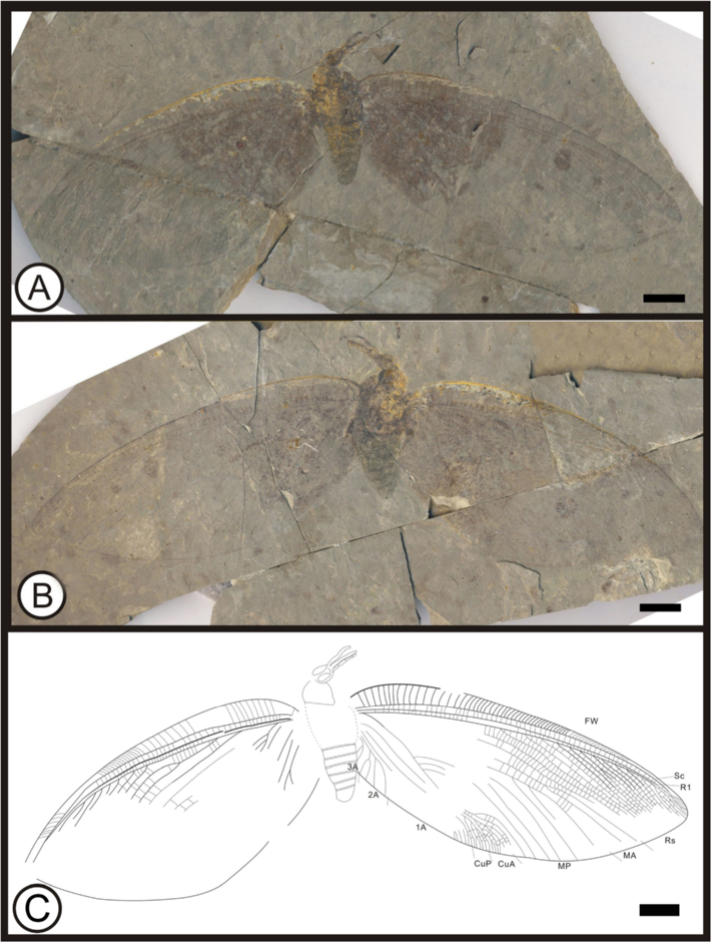
**Images and camera lucida drawing of *Oregramma *sp. CNU-NEU-NN2010-014P. A**, **B**, Habitus; **C**, Outline of habitus. Scale bars, 10 mm.

***Oregramma*** sp. (Figure 15)

#### Diagnosis

Body very robust; head comparatively small. Compound eyes large; siphonate mouthparts present. Forewing without Vr. Rs with ca. 14 primary branches, forked near wing apex. MA bifurcate at vein midsection; MP with more than 4 pectinate branches. 2A with 2 dichotomous branches; 3A bifurcate. Eyespot indistinct.

#### Material

A relatively well-preserved specimen (part and counterpart) with two forewings, and incomplete body; specimen CNU-NEU-NN2010-014P/C (Figure 15A, B). Male.

#### Type locality and horizon

Yixian Formation, Early Cretaceous, Liutiaogou Village, Ningcheng County, Inner Mongolia, China.

#### Measurements

CNU-NEU-NN2010-014P/C (Figure 15A,B): forewing ca. 83 mm long, 34 mm wide as preserved.

#### Description

Robust body preserved in dorsal view (Figure 15C). Head comparatively small, preserved in oblique-lateral view; compound eyes large; antennae not preserved. Mouthparts relatively long; proboscis truncated. Thorax unclear; abdomen of 6 discernible segments. Forewings almost complete, but venation indistinct. Forewing approximately obtuse-triangular. Most costal veinlets curved and not forked, rarely interlinked; Vr absent. Sc and R1 fused apically, then curved posteriorly to enter margin before wing apex. Rs with ca. 14 primary branches, forked immediately before wing apex. MA bifurcate at vein midsection; MP with more than 4 pectinate branches. Posterior wing margin poorly preserved; venation indistinct. CuA and CuP simple, with limited branching at their termini. 1A simple at wing base, but absent along the posterior margin; 2A with 2 dichotomous branches; 3A bifurcate. Inconspicuous eyespot on forewing.

#### Remarks

The specimen is attributed to the genus *Oregramma* based on the above-mentioned characters. Characters defining this specimen are different from other species of the genus. Because of poorly preserved characters, this specimen is unassigned to a species.

Subfamily **Sophogrammatinae** subfam. nov.

(urn:lsid:zoobank.org:act:919EE2ED-DDDA-4D31-9011-F6317FBA2B4A)

**Type genus.***Sophogramma* Ren & Guo, 1996

**Included genera**. Type genus and *Protokalligramma* Yang, Makarkin & Ren, 2011.

#### Diagnosis

Apomorphic characters defining the Sophogrammatinae are: 1), presence of a humeral recurrent vein (Vr); 2), proximal pectinate branching of the first anal vein (1A); and 3), presence of a long, accessory veinlet at the basal part of the media posterior vein (MP2). Plesiomorphically retained characters are: 1), mandibulate mouthparts; 2), absence of wing spots and eyespots; and 3) wings that structurally are highly cantilevered.

#### Remarks

The new subfamily Sophogrammatinae represents the earliest divergence within the Kalligrammatidae, and possesses plesiomorphies that are distinct from other kalligrammatids, namely the MP branches are parallel, while MP branches in other kalligrammatids form a large triangular region; the presence of mandibulate mouthparts versus a siphonate proboscis occurring in all other groups; and the absence of eyespots, while other groups generally have distinct eyespots on the wings.

Genus: ***Sophogramma*** Ren & Guo, 1996

**Type species.***Sophogramma papilionacea* Ren & Guo, 1996

**Included species.** Type species and *Sophogramma eucalla* Ren and Guo, 1996; *Sophogramma lii* Yang, Zhao and Ren 2009; *Sophogramma plecophlebia* Ren and Guo, 1996; and *Sophogramma pingquanica* sp. nov.

**Figure 16:**
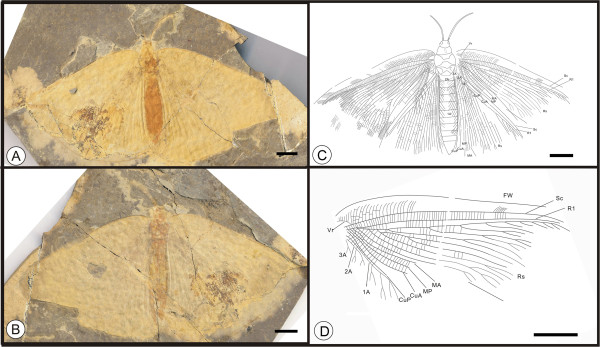
**Images and camera lucida drawings of *Sophogramma pingquanica *sp. nov. CNU-NEU-HP2010-009P. A**, **B**, Holotype; **C**, Outline of Holotype; **D**, Outline of forewing. Scale bars, 10 mm.

***Sophogramma pingquanica*** sp. nov. (Figure 16)

(urn:lsid:zoobank.org:act:7CB0F600-0EC7-4724-B5F2-3747CAAB12D6)

#### Diagnosis

Forewing Vr prominent; third branch of Rs with early dichotomous forking. MA with 3 sinuate and pectinate branches. CuA forked at vein midsection. Hind wing MA forked earlier than all branches of Rs; MP bifurcate at wing base. 2A distinctly dichotomously forked.

#### Etymology

The specific epithet of *pingquanica* is dedicated to the locality of the species in Pingquan County, northeastern China.

#### Holotype

A well-preserved part and counterpart with complete forewings and hind wings, but incomplete body; specimen CNU-NEU-HP2010-009P/C (Figure 16A, B). Sex unknown.

#### Type locality and horizon

Yixian Formation, Early Cretaceous, Pingquan County, Chengde City, Hebei Province, China.

#### Measurements

Holotype, CNU-NEU-HP2010-009P/C (Figure 16A, B): antennae 19 mm long as preserved; forewing 66 mm long, 33 mm as preserved; hind wing ca. 44 mm long, 28 mm wide.

#### Description

Body preserved in dorsal view (Figure 16C). Head partially preserved in dorsal view; compound eyes obvious; antennae filiform, partly preserved, articles unclear. Mouthparts and legs not preserved. Prothorax and thorax indistinct. Hind wings overlapping onto axial body; abdomen with ca. 11 segments discernible. Forewings and hind wings almost complete, but venation unclear.

Forewing approximately obtuse-triangular (Figure 16D); apical area partly missing. Vr present, most of costal veinlets curved and forked. R1 nearly parallel to Sc as preserved; Rs with 11 primary branches; except for the first one, the other branches deeply forked; the third branch (Rs_3_) dichotomously forked earlier. MA with 3 sinuate and pectinate branches, the deeply forked region expansive; MP forked near wing base; MP1 and MP2 forming a loop or cell (bc) basally. CuA forked at the middle of vein; CuP simple but poorly preserved. 1A parallel to CuP for a long distance, with at least 4 pectinate branches; 2A appearing dichotomously forked; 3A simple. Wing eyespot or spot absent.

Hind wing short and broad, nearly acute-triangular. Wing apex absent; most of costal margin hidden by overlap of forewing anal region; C, Sc and crossveins between them barely discernible. Sc and R1 curved posteriorly, R1 simple; Rs with more than 10 branches, each sequentially forked. MA forked earlier than all branches of Rs, with two deeply forked branches; MP bifurcated at wing base. CuA simple; CuP ca. parallel to CuA at posterior part of wing, displaying more than 4 pectinate branches. Anal veins overlapping onto body, margins unclear; 1A with at least 12 pectinate branches; 2A distinctly forked, basal branch missing; 3A not present.

#### Remarks

*Sophogramma pingquanica* sp. nov. is attributed to the genus *Sophogramma* based on the above-mentioned characters. *S. pingquanica* sp. nov. is the second specimen of this genus preserved with four largely separated wings and an intact body, the first being *S. lii*[23]. *Sophogramma pingquanica* sp. nov. is distinguished from other species principally by forewing characters: 1), the third branch (Rs_3_) is dichotomously forked earlier than other congeneric species; and 2), the CuA is forked at the middle of the vein. In addition, the following hind wing features are distinctive: 3), the Rs has more than ten primary branches; 4), the CuP has more than four pectinate branches; and 5), the 1A probably has at least twelve pectinate branches.

### Key to the genera of Kalligrammatidae

The classification of the Kalligrammatidae is principally based on the forewing characters. We omit five genera in the following key: *Angarogramma*[28], and *Palparites*[4], characterized by poor preservation and the lack of sufficient diagnostic details, as well as *Kalligrammina*[16], *Limnogramma*[20], and *Sinokalligramma*[25], with only hind wings preserved.

**1. A**. Wing setae sufficiently dense that veins are obscured (Figure 11, 12).………………………………………………….. *Ithigramma* gen. nov.

**B**. Wing setae relatively sparse, veins quite evident........................................................ **2**

**2. A**. Forewing of simple costal veinlets simple with few distal forks................................. **3**

**B**. Forewing of complex costal veinlets, with many distal forks..................................... **4**

**3. A**. MP1 and MP2 forming a loop or cell (bc) (Figures one–three in [21]) basally........ *Sophogramma*

**B**. Absence of the loop or cell (bc) between MP1 and MP2............................................ **5**

**4. A**. Presence of ORB between Rs and MA (Figure two in [22])............................................... **6**

**B**. Absence of ORB between Rs and MA........................................................................ **7**

**5. A**. Many rows of basal cells between CuA & CuP and 1A & 2A (Figure 10)............................. *Abrigramma* gen. nov.

**B**. Only one row of basal cells between CuA & CuP and 1A & 2A................................ **8**

**6. A**. Two ORBs present near wing base between Rs and MA

(Figure 1)………………………………………………………… *Affinigramma* gen. nov.

**B**. Many ORBs present along the main stem of Rs......................................................... **9**

**7. A**. First branch of Rs distal from wing base (Figure three in [11]).................... *Meioneurites*

**B**. First branch of Rs proximal to wing base................................................................. **10**

**8. A**. MP sinuate (Figure 13).......................................................... *Oregramma*

**B**. MP rectilinear (Figure twenty-six in [15])............................................. *Lithogramma*

**9. A**. More than twenty ORBs; forewing with distinct spots

(Figures four–five in [24])................................................................ *Apochrysogramma*

**B**. Ten or less ORBs; forewing without distinct spots............................ *Kallihemerobius*

**10. A**. MP forming a large triangular region........................................................................ **11**

**B**. MP stem simple, dichotomously forked (Figures one–two in [24])......... *Protokalligramma*

**11. A**. Presence of humeral recurrent vein (Figure 5).................................................. **13**

**B**. Absence of humeral recurrent veins.......................................................................... **12**

**12. A**. CuA and CuP forked proximally (Figure twenty-seven [15])............. *Kalligrammula*

**B**. CuA and CuP always forked distally....................................................... *Kalligramma*

**13. A**. MP pectinately forked (Figure 5)…………. *Stelligramma* gen. nov.

**B**. MP dichotomously forked (Figure two in [27])……............................. *Huiyingogramma*

## Discussion

### Results of the phylogenetic analysis

An analyses using NONA with four successive outgroups resulted in nine most parsimonious trees (MPTs), each consisting of 78 steps (CI = 0.65, RI = 0.82). Species in the same genus were well grouped in the cladogram except for *Ithigramma* and *Oregramma* (Additional file 1: Figure S1A-D). The genus *Ithigramma* with its character of densely setose forewings distinctly differed from *Oregramma*. However, *Ithigramma* was nested within an *Oregramma* clade in the MPTs. This paraphyly probably was caused by poor preservation of the *Ithigramma* specimen, which lacked sufficient information for differentiation from *Oregramma*. In addition, the other genera were well grouped monophyletically in the analysis with a minor exception in the interrelationships of *Kalligramma* (Figure S1).

The analyses by PAUP resulted in 40 MPTs, also of 78 steps (CI = 0.65, RI = 0.82), which were similar to the NONA analyses. In both trees of strict consensus and 50% major consensus, *Ithigramma* and *Oregramma* became a paraphyly, with slightly different results vs. the NONA analyses (Additional file 2: Figure S2).

The Bayesian analyses did not adequately resolve the interrelationships of the Kalligrammatidae (Additional file 3: Figure S3), similar to the parsimony analyses. In the Bayesian analyses, the Kalligrammatidae was divided into three paraphyletic groups; however, the clade *Kalligramma* was not recovered as a monophyly (Additional file 3: Figure S3), same as the results of parsimony analyses. The other apparent discrepancy between Bayesian and parsimony analyses (Additional file 4: Figure S4) involved the placement of *Sophogramma*, grouped with *Kalligrammula*, *Stelligramma*, *Lithogramma, Affinigramma, Huiyingogramma* and *Kallihemerobius*, instead of the earliest divergence of the Kalligrammatidae. However, there are some clades in which Bayesian and parsimony analyses agree, confirming three monophyletic groups: *Sophogramma*, *Meioneurites*, and an *Abrigramma* + *Ithigramma* + *Oregramma* clade.

### Phylogeny of Kalligrammatidae

**Figure 17:**
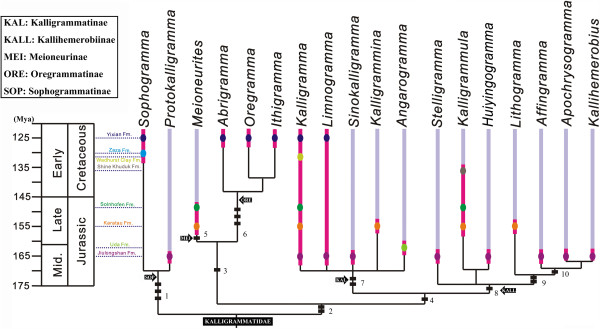
**A composite system of the phylogenetic relationships within the Kalligrammatidae. 1**: **a**, Parallel MP vein branches, **b**, Eyespot absence, **c**, Presence of a humeral recurrent vein (Vr). **2**: **a**, A large triangular MP region, **b**, Presence wing eyespots. **3**: Simple costal crossveins, with few interlinked veinlets. **4**: **a**, Complex costal cross-venation, **b**, Deep MA forks. **5**: Distal branching of MP. **6**: **a**, Distal constriction of the costal region; **b**, Sinuate MP; **c**, Expanded region between CuA and CuP. **7**: **a**, Broadly triangular forewing; **b**, 1A bifurcation at wing midsection or distally. **8**: Proximal bifurcation of 1A vein. **9**: **a**, Presence of a broad costal region; **b**, Few Rs branches. **10**: Presence of an ORB.

Because of incomplete information from a few poorly preserved specimens, not all clades received firm support in the phylogenetic analyses. Based on the phylogenetic results (Additional file 4: Figure S4A, B), we propose a rationalized phylogenetic tree of the Kalligrammatidae that includes all genera, but excludes *Palparites* (Figure 17). In the phylogenetic analysis, the monophyly of the Kalligrammatidae is firmly recovered, which shares the synapomorphic characters of a complex MA bifurcation and a broad triangular forewing and hind wing. The Kalligrammatidae is divided into five primary clades, allocated to the subfamilies Sophogrammatinae subfam. nov., Meioneurinae subfam. nov., Oregrammatinae subfam. nov., Kalligrammatinae [4] and Kallihemerobiinae [29].

The assemblage of the Sophogrammatinae subfam. nov. was expected, which contains two well-defined genera, *Sophogramma*[21] and *Protokalligramma*[24]. However, the phylogenetic position of the subfamily remains in question because of the difference between the parsimony and Bayesian analysis. We note that the clade consisting of the Sophogrammatinae and Kallihemerobiinae received low posterior probability in the Bayesian results (Additional file 4: Figure S4B). The grouping of the Sophogrammatinae and Kallihemerobiinae is likely an artificial construct from incomplete data. Based on the parsimony results, the Sophogrammatinae represents the earliest divergence of the Kalligrammatidae, and is the sister-group to all other kalligrammatids. This subfamily can be differentiated from other subfamilies by the trajectory of the MP, in which both branches are parallel and bent posteriorly and distally, versus the alternate condition in which the MP_1_ diverges from the MP_2_ close to the wing base, and forms a large triangular region, present in the other four subfamilies (Figure 17/node 1). However, the absence of eyespots within the Sophogrammatinae [*Sophogramma*[21,23] and *Protokalligramma*[24]] distinctly implies a plesiomorphy for Kalligrammatidae (Figure 17/node 1); hence, the occurrence of eyespots in other kalligrammatids should be a derived feature. Nevertheless, the absence of eyespots among *Kalligrammula*, *Meioneurites spectabilis* and *Angarogramma* is likely the result of poor preservation or possibly plesiomorphous retention. The humeral recurrent vein is slightly variable within most Kalligrammatidae, whereas it is well represented in the Sophogrammatinae and can be regarded as a synapomorphy of the subfamily (Figure 17/node 1).

The other four subfamilies share the three synapomorphic characters (Figure 17/node 2) of eyespot presence, and a distinctively triangular MP region. This larger clade is subdivided into two clades, the Meioneurinae subfam. nov. + Oregrammatinae subfam. nov., and the Kalligrammatinae [4] + Kallihemerobiinae [29] (Figure 17/node 3, 4). The sister-group relationships of the Meioneurinae and Oregrammatinae are stable in the phylogenetic analysis, and weakly supported by the two homoplasious characters of less interlinked veinlets along the costal region and simple distal Cu1 forks. The subfamily Meioneurinae consists of only one genus, *Meioneurites*[4], from the Late Jurassic, and has the character of distal branching of MP (Figure 17/node 5). The subfamily Oregrammatinae includes three highly homogenous genera: *Oregramma*[20], *Abrigramma* gen. nov., and *Ithigramma* gen. nov., that share the three synapomorphic characters of a distally constricted costal region, a sinuate MP close to the wing base and a medially broadened Cu region (Figure 17/node 6).

Although the Kalligrammatinae and Kallihemerobiinae are not grouped as a clade in the Bayesian analyses, we prefer the sister relationship of the Kalligrammatinae and Kallihemerobiinae that was firmly supported by the character of a deep MA bifurcation in the parsimony results (Figure 17/node 4). In the analyses, the iconic genus *Kalligramma* was not fully recovered from the Bayesian results (Additional file 3: Figures S3, Figure 4). However, *Kalligramma* is well established by the single synapomorphic character of a distal complex CuA bifurcation in the parsimony analysis (Additional file 1: Figures S1, Figure 2). We consider the controversial placement of this genus might be caused by poor preservation of the specimens. Herein, we tentatively assign four other kalligrammatine-like genera – *Angarogramma*[28], *Limnogramma*[20], *Kalligrammina*[16] and *Sinokalligramma*[25] – to the subfamily Kalligrammatinae based on the presumed characters of a broadly triangular forewing and hind wing, and a 1A vein forked medially or distally (Figure 17/node 7). The interrelationships of Kalligrammatinae currently are not fully resolved because of an inordinate amount of missing data.

In the phylogenetic results the monophyly of Kallihemerobiinae is partially recovered, principally based on sharing the single plausible character of a proximal bifurcation of the 1A vein, retrieved from the parsimony analysis (Figure 17/node 8). Based on the results of our analysis, we expand the definition of the Kallihemerobiinae. As for the former definition of the subfamily based on the presence of the ORB, only three genera, *Affinigramma*, *Apochrysogramma* and *Kallihemerobius*, can be attributed to the Kallihemerobiinae (Figure 17/node 10). The affiliation of the four heterogeneous genera of *Stelligramma, Kalligrammula, Huiyingogramma* and *Lithogramma* to the subfamily is not consistent with our current knowledge, which is probably the result of poor preservation for specimens of these genera. The genus *Lithogramma* seems to be related to the traditional Kallihemerobiinae, sharing a similar expanded costal region and fewer Rs branches (Figure 17/node 9). The genera *Huiyingogramma, Kalligrammula* and *Stelligramma* are attributed to Kallihemerobiinae temporarily, pending future additional evidence to determine their subfamilial status.

Although the interrelationships among the subfamilies are not fully resolved, our analyses are an initial probe into the internal phylogeny of Kalligrammatidae. Future fossil discoveries and additional structural evidence, as they become available, as well as new analyses, will further a more secure placement of the less character-rich taxa.

### Diversity of the Kalligrammatidae

**Figure 18:**
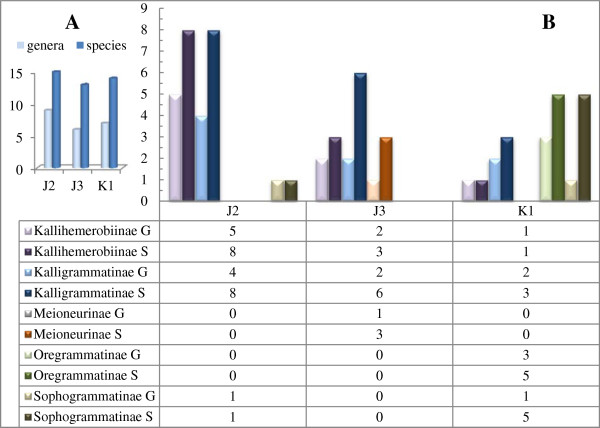
**Histograms of kalligrammatid species and generic diversity during their 40 million-year existence during the mid Mesozoic. A**, Histogram showing the genus and species richness of the Kalligrammatidae. **B**, Histogram showing the genus and species richness at the subfamilial level within the Kalligrammatidae.

The Kalligrammatidae were a dominant lineage among Neuroptera during the Mesozoic Era, as measured by speciosity. From the histograms of species and generic richness (Figure 18A), kalligrammatids were well established and exhibited, as a group, a relatively flat level of diversity from the late Middle Jurassic at 165 Ma to the mid Early Cretaceous at 125 Ma., and are recorded from important Eurasian deposits. However, the internal composition of taxa does vary (Figure 18B). The diversity of the subfamily Kallihemerobiinae, for example, gradually decreases from the later Middle Jurassic to the earlier Early Cretaceous. A similar pattern also was present for the subfamily Kalligrammatinae, abundant during the Middle Jurassic and Late Jurassic, but slackening considerably during the Early Cretaceous. The two sister subfamilies of Meioneurinae and Oregrammatinae occur solely during the Late Jurassic and Early Cretaceous, respectively, revealing the independent origin of new phylogenetic lineages long after earlier kalligrammatid lineages were established during the earlier Jurassic. The subfamily Sophogrammatinae was recorded from two separate periods: the Middle Jurassic, where it probably represents the most basal kalligrammatid lineage, and the Early Cretaceous, implying a temporally displaced rediversification of the subfamily.

**Figure 19:**
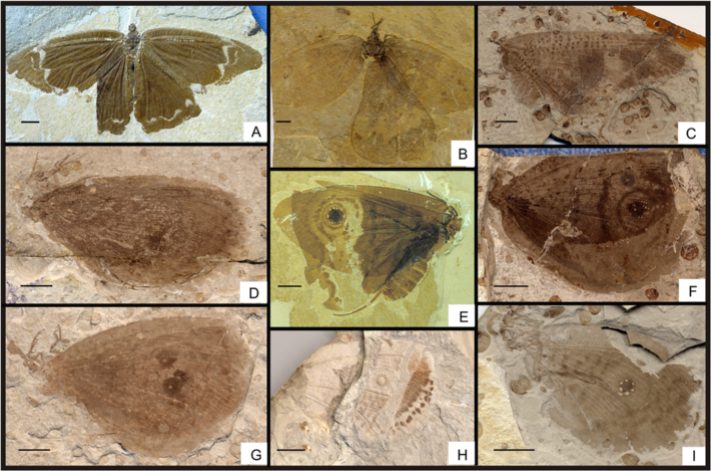
**Structural diversity among Kalligrammatidae. A**, *Sophogramma lii* Yang, Zhao and Ren, 2009. **B**, *Oregramma aureolosa* sp. nov. **C**, *Stelligramma allochroma* sp. nov. **D**, *Affinigramma myrioneura* sp. nov. **E**, *Oregramma illecebrosa* sp. nov. **F**, *Kalligramma circularia* sp. nov. **G**, *Affinigramma myrioneura* sp. nov. **H**, *Apochrysogramma rotundum* Yang, Makarkin and Ren, 2011. **I**, *Kalligramma brachyrhyncha* sp. nov.

As for most neuropterans, kalligrammatids probably were weak flyers, and the typically large body size undoubtedly made them easy targets for contemporaneous predators. The same condition also occurred in other Mesozoic groups of the Neuroptera, the extinct Aetheogrammatidae [29], Saucrosmylinae [33], Grammolingiidae [34] and Panfiloviidae [35], all of which possessed rather large body size. For survival and reproduction, these large insects required special avoidance strategies. Wang et al. [36] reported that the earliest and only known pinnate leaf mimesis by lacewings was *Bellinympha filicifolia*[36] and *Bellinympha dancei*[36], both from the late Middle Jurassic. This association exhibited unique behavioral features, revealing a specialized association between insects and gymnosperms that was lost in more recently derived lineages. As for the Kalligrammatidae, the group presents several morphological and behavioral innovations (Figure 19). The presence of eyespots in the Kalligrammatidae implies that kalligrammatids were probably diurnal insects. *Sophogramma lii* possesses two symmetrical, undulate light-colored stripes near the outer wing margin [23], which can be interpreted as a disruptive coloration (Figure 19A). In a related phenomenon, Olofsson et al. [37] reported wing-margin eyespots in a lepidopteran that reduced attacks on vital body parts by birds. The marginal markings on *Sophogramma lii* likely had a similar defensive function, misleading predators to attack the wing margin instead of vital, axially located body parts.

During the 40 million years from the late Middle Jurassic (165 Ma) to mid Early Cretaceous (125 Ma), the Kalligrammatidae may have provided an important role in their relationships with plants and avoidance of predators. Kalligrammatids bore either mandibulate or more commonly siphonate mouthparts, often deployed wing markings, including stripes, spots and eyespots and displayed varied wing shapes, indicating that the Kalligrammatidae underwent an evolutionary diversification during the mid-Mesozoic. The disappearance of Kalligrammatidae and absence of their diverse features in modern neuropterans is enigmatic, although there may be modern analogs [2,38]. The eventual extinction of these giant insects may have been precipitated by the emergence of angiosperms and associated environmental turnover that began during the mid-Early Cretaceous.

## Conclusions

New fossils from China provide insight into the evolution of the Kalligrammatidae, and suggest that Eastern Eurasia may have been the center of origin for the family during the mid-Mesozoic. Based on phylogenetic analyses and detailed morphological characters, we describe several new genera and species, and propose a new classification encompassing the Kalligrammatidae that is partitioned into five principal clades that are accorded subfamilial rank. They are Sophogrammatinae subfam. nov., Meioneurinae subfam. nov., Oregrammatinae subfam. nov., Kalligrammatinae [4] and Kallihemerobiinae [29]. Kalligrammatids are perhaps the most successful Mesozoic neuropteran lineage, exhibiting an extraordinary breadth of form, taxonomic diversity and ecological dominance that included a variety of plant-insect associations and deterrence from predators. These features indicate that the evolutionary biology of mid Mesozoic kalligrammatid lacewings was more complex than previously realized.

## Methods

The specimens were examined under a Leica MZ12.5 dissecting microscope (Leica, Wetzlar, Germany). Line drawings were prepared with CorelDraw 12 graphic software with the assistance of Photoshop CS2 (Adobe Systems, Mountain View, California). The photographs were taken by Epson Perfection 1650 and Nikon D100 digital cameras. Magnified images of key specimen areas were taken with a Nikon SMZ1000 stereo microscope. All type materials described in this report are deposited in the Key Lab of Insect Evolution and Environmental Changes, College of Life Sciences, Capital Normal University, in Beijing, China (CNUB; Dong Ren, Curator). The LSID for this publication on ZooBank is: urn:lsid:zoobank.org:pub:1CDD4A8D-E8FB-4FE8-AF1E-3051B9273EC1.

We conducted a phylogenetic analysis using morphological data from 27 comparatively character-rich specimens of kalligrammatids and related outgroups. Eleven specimens are newly described and sixteen are from the literature. The phylogenetic analysis was overwhelmingly (28 of 30) based on forewing characters (Additional file 5), as kalligrammatid taxa are fundamentally erected on forewing vein structure. Ten genera of the Kalligrammatidae, including 23 species, were sampled in the analysis. Because some previously described species consisted only of hind wings, or information was minimal from the original descriptions, some taxa were too limited to be adequately scored. Seventeen other species were omitted in the analysis (Additional file 6: Table S1). With respect to the peculiar venation of the Kalligrammatidae, four representative genera in other neuropteran families were selected as outgroups, previously reported as closely related to Kalligrammatidae: *Saucrosmylus* (Osmylidae) [33], *Panfilovia* (Panfiloviidae) [16], *Aetheogramma* (Aetheogrammatidae) [29] and *Grammolingia* (Grammolingiidae) [34].

The matrix consists of 28 taxa and 30 morphological characters and their character states, displayed in Additional file 7: Table S2. The taxa–character matrix was edited using a NEXUS Data Editor (version 0.5.0). All characters were treated as unordered states, and weighted equally. The tree search was conducted by two different inference strategies, parsimony analyses and Bayesian analyses, in order to find preferred cladograms. In parsimony analyses, two phylogeny-seeking programs were employed: WinClada/NONA, and PAUP version 4.0b10. The data matrix was subjected to NONA [39] analyses, employing a heuristic parsimony analysis, with options set to hold 10000 trees, perform 1000 replications with one starting tree replication, and use of a multiple TBR + TBR search strategy. Because NONA can use only one defined outgroup for each analysis, we conducted the cladistic analyses by using each of the four outgroups in successive searches. In PAUP, parsimony analyses were conducted using the branch-and-bound algorithm [40]. Bremer decay indices were obtained using command files composited by TreeRot, version 3 [41], in conjunction with the heuristic search algorithm in PAUP version 4.0b10.

Bayesian inference analyses were conducted with MrBayes version 3.1.2 [42,43]. The maximum likelihood setting referred to the discrete morphological model developed by Lewis [44]. The morphological data were modeled under the assumption that only the variable characters among taxa were included, and gamma-shaped rate variation was enforced. Prior probabilities were kept at their default settings for standard (morphological) analyses. Each analysis was run for 10^6^ generations. Samples were taken every 10^2^ generations, resulting in a total of 10^4^ samples for each of the parallel analyses. The first 2.5 × 10^3^ samples were discarded, representing the “burn-in” period. As with NONA, MrBayes 3.1.2 also was limited to using one defined outgroup for each analysis, which was implemented using each outgroup successively.

## Competing interests

The authors declare that they have no competing interests.

## Authors' contributions

QY, YJW, CCL, CKS carried out fossil processing, photography, figure preparation, data analysis and interpretation, drafting of manuscript and completion of study. DR conducted fieldwork, specimen collection, data analysis, and manuscript review and revision. All authors read and approved the final manuscript.

## Supplementary Material

Additional file 1: Figure S1 50% majority-rule consensus tree of 9 MPTs from NONA. **A**, *Saucrosmylus* assigned as outgroup; **B**, *Panfilovia* assigned as outgroup; **C**, *Grammolingia* assigned as outgroup; **D**, *Aetheogramma* assigned as outgroup. Numbers represent percentage support values. Click here for file

Additional file 2: Figure S2 Phylogenetic results from PAUP. **A**, Strict consensus tree, The Bremer decay index is indicated at each branch; **B**, 50% majority-rule consensus tree of 40 MPTs by PAUP. Click here for file

Additional file 3: Figure S3 Phylogenetic trees from Bayesian analyses. **A**, *Saucrosmylus* assigned as outgroup; **B**, *Panfilovia* assigned as outgroup; **C**, *Aetheogramma* assigned as outgroup; **D**, *Grammolingia* assigned as outgroup. Values associated with nodes indicate posterior probabilities. Click here for file

Additional file 4: Figure S4 Comparison between parsimony and Bayesian results. **A**, The best supported tree of the most parsimonious trees. **B**, The Bayesian tree. Click here for file

Additional file 5 List of characters and character states for phylogenetic analysis. Click here for file

Additional file 6: Table S1 Genera and species omitted in the analyses. Click here for file

Additional file 7: Table S2 Taxon–character-state matrix. Click here for file
