# Supplementary figures and images for: Mesozoic lacewings from China provide phylogenetic insight into evolution of the Kalligrammatidae (Neuroptera)
Source: BMC Evol Biol. 2014 Jun 9;14:126. doi: 10.1186/1471-2148-14-126 (PMC4113026; doi:10.1186/1471-2148-14-126)

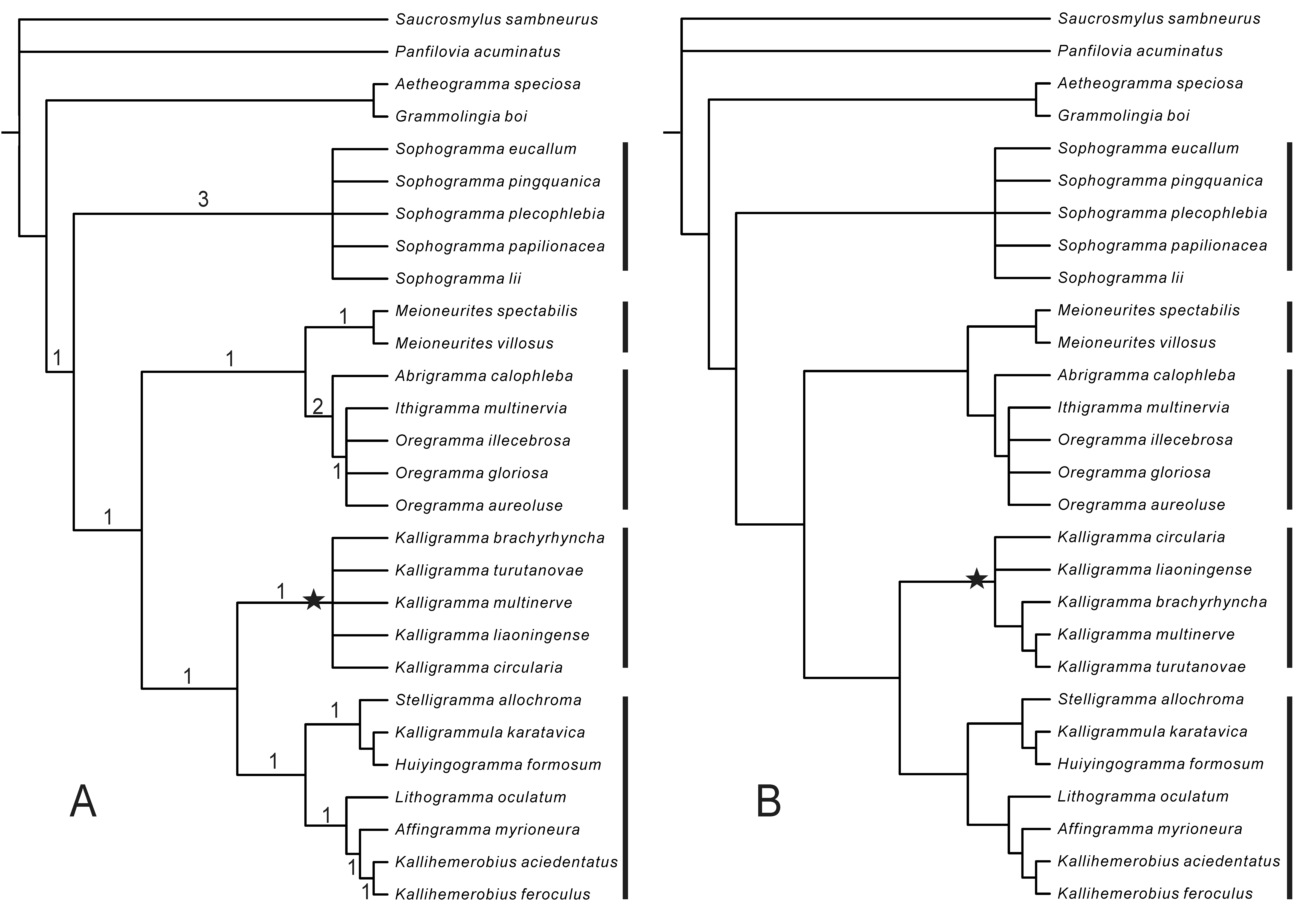

Supplement: Additional file 2: Figure S2 — Phylogenetic results from PAUP. A, Strict consensus tree, The Bremer decay index is indicated at each branch; B, 50% majority-rule consensus tree of 40 MPTs by PAUP. [file 1471-2148-14-126-S2.tif]

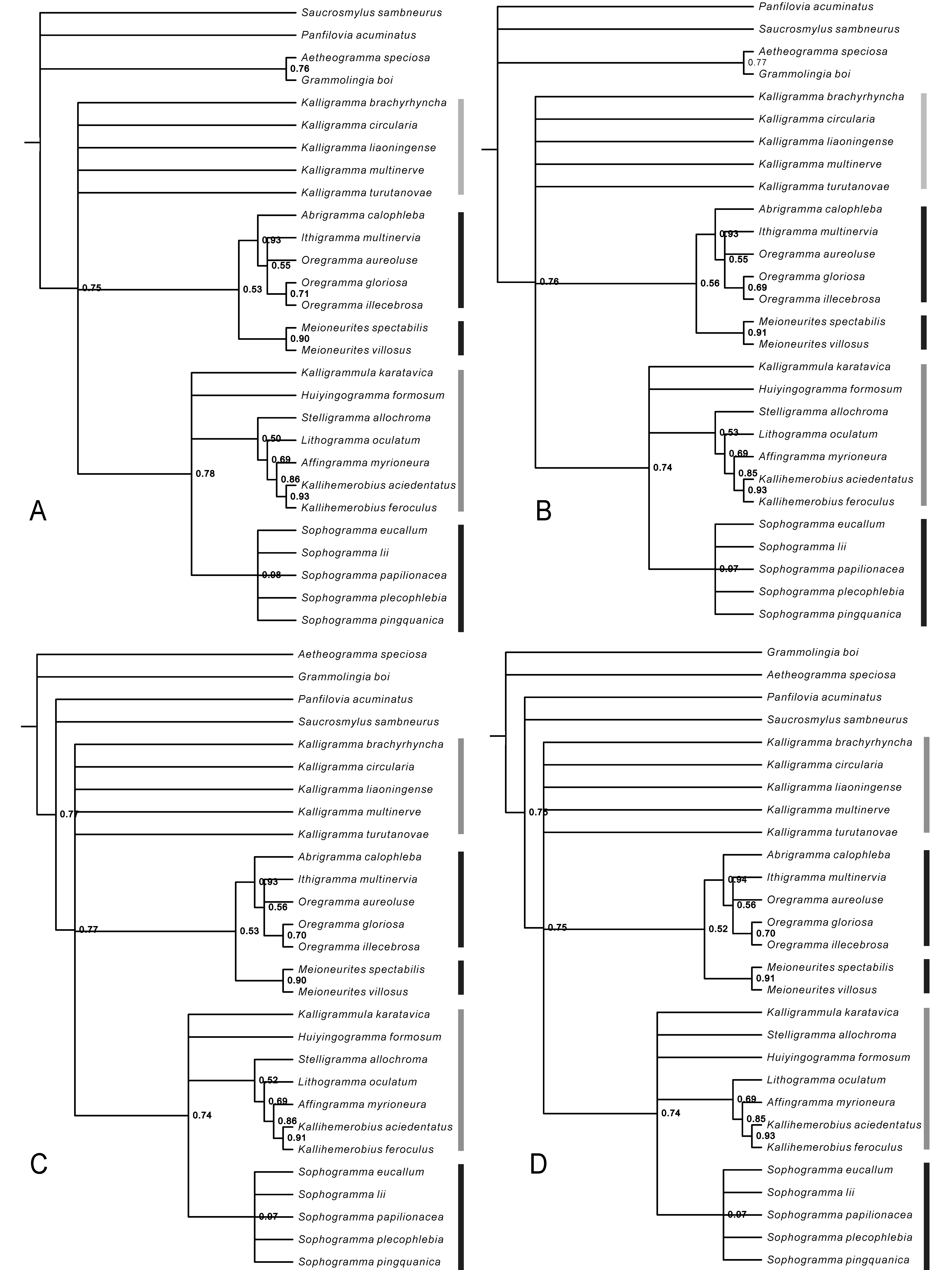

Supplement: Additional file 3: Figure S3 — Phylogenetic trees from Bayesian analyses. A, Saucrosmylus assigned as outgroup; B, Panfilovia assigned as outgroup; C, Aetheogramma assigned as outgroup; D, Grammolingia assigned as outgroup. Values associated with nodes indicate posterior probabilities. [file 1471-2148-14-126-S3.tif]

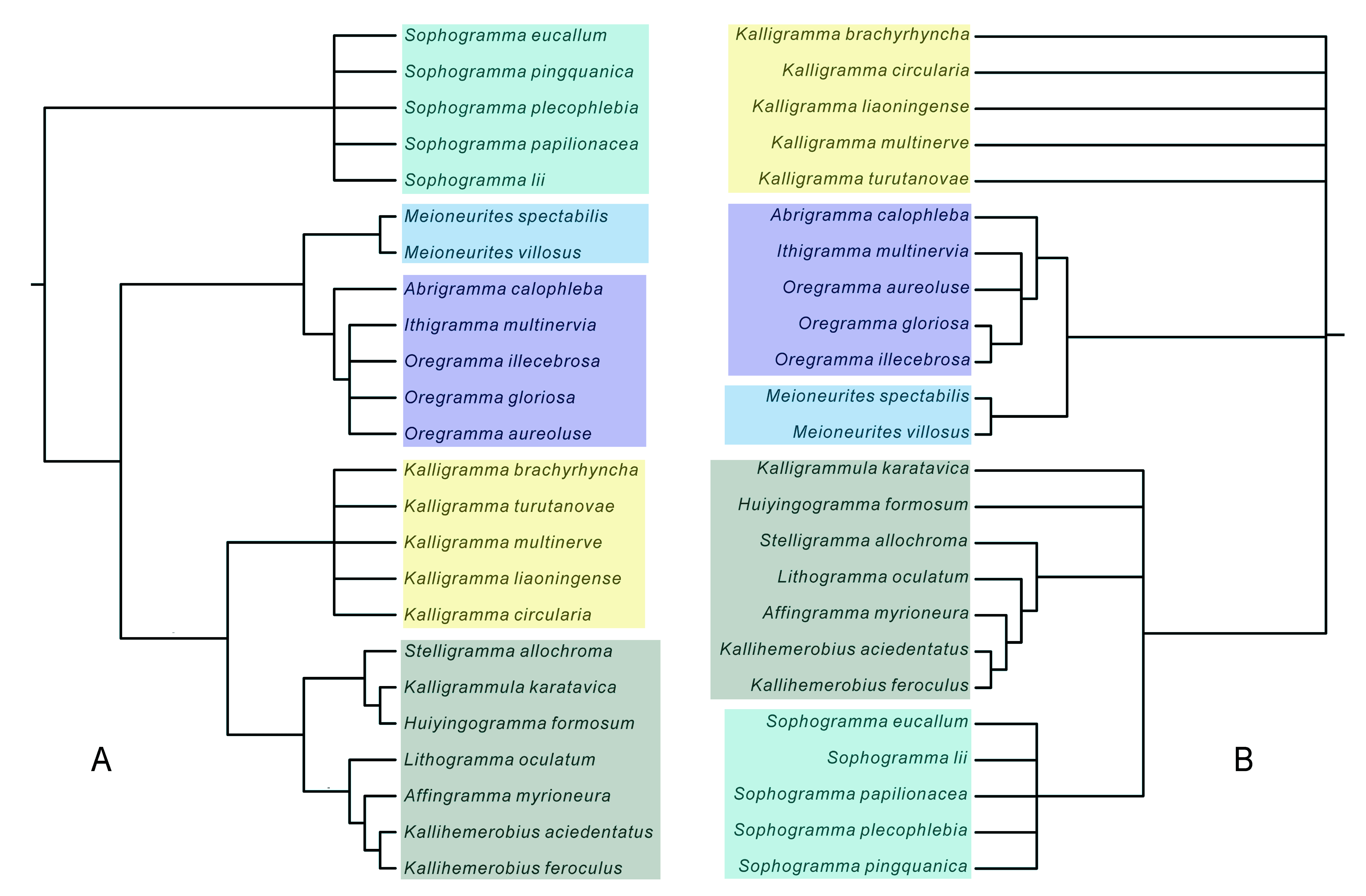

Supplement: Additional file 4: Figure S4 — Comparison between parsimony and Bayesian results. A, The best supported tree of the most parsimonious trees. B, The Bayesian tree. [file 1471-2148-14-126-S4.tif]
