# Supplementary material for: Mesozoic lacewings from China provide phylogenetic insight into evolution of the Kalligrammatidae (Neuroptera)
Source: BMC Evol Biol. 2014 Jun 9;14:126. doi: 10.1186/1471-2148-14-126 (PMC4113026; doi:10.1186/1471-2148-14-126)
Supplement: Additional file 5 — List of characters and character states for phylogenetic analysis. [file 1471-2148-14-126-S5.docx]

**List of characters and character states for phylogenetic analysis**

1. **Humeral recurrent vein (Vr).** (**0**), absent; (**1**), present. The humeral vein commonly exhibits a simple branch, as State (0), in most Neuroptera, and is considered a plesiomorphy. State (1), with a recurrent branch, occurs in the kalligrammatid genera *Stelligramma* and *Sophogramma*.

2. **Size of costal region.** (**0**), gradually narrowed; (**1**), strongly constricted close to the wing apex (*Abrigramma*, *Ithigramma*, *Oregramma*); (2), remarkably expanded (occurring only in *Affinigramma*, *Lithogramma* and *Kallihemerobius*).

3. **Interlinked veinlets between costal crossveins.** (**0**), absent; (**1**), present, forming 1–2 rows of gradational series; (**2**), present, complex and forming more than two rows of a gradational series. State (0) occurs in the outgroup *Aetheogramma*, and in three genera of Kalligrammatidae: *Ithigramma*, *Meioneurites* and *Oregramma*. State (1) is inferred as being derived from state (0), occurring in the outgroup *Grammolingia* and in one species of *Kalligramma*. State (2) occurs in most kalligrammatid species.

4. **Costal crossveins.** (**0**), simple or with few distal forks; (**1**), with numerous distal forks; (**2**), with complex bifurcating forks. State (0) consists of simple costal crossveins and is common to the Neuroptera, occurring in the outgroups *Aetheogramma* and *Grammolingia*, and the kalligrammatids *Ithigramma* and *Oregramma*. State (1) occurs in two other outgroups, *Saucrosmylus*, *Panfilovia*, but also is present in most kalligrammatid species. Complex costal crossveins are considered as an autapomorphy of the kalligrammatids *Affinigramma* and *Kallihemerobius*.

5. **Distal part of Sc and R1.** (**0**), Sc and R1 dissociated at the distal wing area; (**1**), Sc and R1 fused distally. The dissociation of the distal part of Sc and R1 is primitive, occurring in the outgroups *Aetheogramma* and *Grammolingia*. The fusion of Sc and R1 is present in most kalligrammatid species.

6. **Position of Sc and R1 entering the wing margin.** (**0**), entering the margin before the apex; (**1**), entering the margin beyond the apex. State (0) is common for Neuroptera and is regarded as a plesiomorphy. State (1) occurs in the outgroups *Aetheogramma* and *Grammolingia*, and in the Kalligrammatidae.

7. **Number of sc–r1 crossveins.** (**0**), one; (**1**), numerous. A single sc–r1 crossvein is a plesiomorphy. Dense sc–r1 crossveins occur in the outgroups *Panfilovia*, *Aetheogramma* and *Grammolingia*, and in the Kalligrammatidae.

8. **R1 distal branch.** (**0**), shallow forking present; (**1**), deep forking present. Shallow forking in the R1 distal branch is the primitive State (0). State (1) is an autapomorphy of the outgroup *Aetheogramma*.

9. **Rs stem.** (**0**), originating close to the wing base, nearly as long as R1; (**1**), originating distant from the wing base; (**2**), originating near the divergence of R1. The origin of the Rs stem proximal to the wing base is a plesiomorphy. States (1) and (2) are inferred to derive from State (0). State (2) is considered as an autapomorphy of the kalligrammatid *Kallihemerobius*.

10. **Number of Rs branches.** (**0**), less than 10; (**1**), more than 9. The number of Rs primary branches is variable among species; however, it is relatively stable at the generic level. State (0) is found in the outgroups *Aetheogramma* and *Grammolingia*, and in the kalligrammatids *Kallihemerobius*, *Kalligrammula*, *Lithogramma* and *Meioneurites*. State (1) occurs in other kalligrammatid genera.

11. **Rs branch.** (**0**), only with distal, shallowly forking branches; (**1**), with deeply forking branches. Shallow forking of the Rs branches is common in Neuroptera and is assigned a plesiomorphy. State (1) occurs in five genera of Kalligrammatidae: *Affinigramma*, *Kallihemerobius*, *Kalligrammula*, *Lithogramma* and *Sophogramma*.

12. **Configuration of Rs distal branches.** (**0**), straight; (**1**), bent. State (0) is common in Neuroptera and is referred to a plesiomorphy. Bent Rs branches are found in one species of *Kalligramma* and in *Sophogramma*.

13. **Oblique radial branches (ORB).** (**0**), absent; (**1**), present, 1–2 branches; (**2**), more than 2 branches. The true Rs originates from the R1 and forms many pectinate branches in most Neuroptera. Oswald (33) introduced a new term ORB (oblique radial branch) to interpret the special venation of the Hemerobiidae. Herein, we adapt his term to interpret the venation of *Affinigramma* and *Kallihemerobius*. The true Rs of *Kallihemerobius* is distant to the wing base, and there are some oblique radial branches (ORB) evident in State (2).

14. **MA forking.** (**0**), shallow branching; (**1**), distal pectinate branching; (**2**), distal dichotomous branching; (**3**) deep branches, close to the midvein. Shallow branching appears to be plesiomorphic. State (1) is restricted to three kalligrammatid genera: *Meioneurites*, *Oregramma* and *Sophogramma*. State (2) emerges in *Kalligramma*, *Kalligrammula* and *Huiyingogramma*. State (3) present in the outgroup *Panfilovia*, and the five kalligrammatid genera: *Abrigramma*, *Affinigramma*, *Kalligramma*, *Kallihemerobius* and *Lithogramma*.

15. **MP forking.** (**0**), parallel branching; (**1**), triangularly branching. Parallel MP branches appear to be plesiomorphic in Neuroptera and restricted to outgroups and to *Sophogramma*. State (1) only emerges in the outgroup *Aetheogramma* and in other kalligrammatid taxa.

16. **Basal portion of MP.** (**0**), originates from the wing base or is a short stem; (**1**), is a long stem. The basal fork of the MP is common to Neuroptera and occurs in most species. State (1) emerges in *Meioneurites* and represents an autapomorphy of the genus.

17. **MP1 forking.** (**0**), shallow branching; (**1**), deep branching. Shallow MP1 branches are plesiomorphic; by contrast, deep branches are derived and serve as an autapomorphy of the species *Kalligramma turutanovae*.

18. **Configuration of MP2.** (**0**), straight; (**1**), arched basally. A straight MP2 is plesiomorphic whereas a basally arched MP2 is derived from State (0). State (1) is present in the outgroup *Aetheogramma* and in the kalligrammatids *Abrigramma* and *Oregramma*.

19. **Basal part of MP2.** (**0**), simple; (**1**), with a long accessory veinlet. State (1) occurs in most species of Neuroptera, and is assigned as a plesiomorphy. State (1) occurs solely in the kalligrammatid genus *Sophogramma*.

20. **MP cells.** (**0**), a single row; (**1**), multiple rows. A single row of cells between the MP branches appears to be plesiomorphic; State (1) occurs only in the kalligrammatid *Abrigramma* and serves as an apomorphy for the genus.

21. **Cu region.** (**0**), normal, parallel; (**1**), broadened along the midveinal interval. State (0) is common to Neuroptera; State (1) occurs in the kalligrammatids *Abrigramma*, *Ithigramma* and *Oregramma*.

22. **CuA forking.** (**0**), distal, simple branches; (**1**), distal, complex branches; (**2**), dichotomously branched at midvein; (**3**), forked at midvein and forming many pectinate branches; (**4**), branching along the proximal vein course. Simple branching along the distal CuA is regarded plesiomorphic and evolved into the other character-states.

23. **CuP forking.** (**0**), distal simple branches; (**1**), distal complex branches; (**2**), dichotomously branched in the middle; (**3**), branching along the proximal portion of the vein.

24. **A1 forking.** (**0**), distal simple branches; (**1**), distal pectinate branches; (**2**), deeply forked branches, but not beyond the midvein segment; (**3**), deep branches, close to the A1 base; (**4**), proximal, pectinate branches.

25. **Surface of forewing.** (**0**), glabrous or moderately hairy; (**1**), densely hirsute.

26. **Shape of forewing.** (**0**), elongate or oblong; (**1**), triangular, approximately symmetric about a horizontal axis; (**2**), broadly triangular.

27. **Wing eyespot or spot.** (**0**), absent; (**1**), present, a simple spot; (**2**), present, eyespot enveloped with one or two circular rings; (**3**), present, displaying a few to many ocules (small, ovoidal, whitish areas). Eyespot and spots are absent in most families of Neuroptera, and are considered an apomorphy of the Kalligrammatidae.

28. **Shape of hind wing.** (**0**), elongate or oblong; (**1**), triangular, approximately symmetric about a horizontal axis; (**2**), pyriform.

29. **Ovipositor.** (**0**), absent or inconspicuous; (**1**), present, sword shaped.

30. **Mouthparts.** (**0**), Mandibulate mouthparts with short palpi; (**1**), stout, setose palpi longer than the siphonate proboscis; (**2**), elongate siphonate mouthparts with palpi at approximately the same length as or shorter than the proboscis; (**3**), siphonate mouthparts with palpi demonstrably longer than proboscis. Chewing mouthparts are plesiomorphic to Neuroptera, occurring in outgroups and in the kalligrammatid *Sophogramma*. State (1) emerges in two genera: *Abrigramma* and *Meioneurites*. State (2) occurs in *Affinigramma*, *Kallihemerobius*, *Kalligramma*, *Oregramma* and *Stelligramma*. State (3) is found only in *Ithigramma*.
