# Supplementary material for: Mesozoic lacewings from China provide phylogenetic insight into evolution of the Kalligrammatidae (Neuroptera)
Source: BMC Evol Biol. 2014 Jun 9;14:126. doi: 10.1186/1471-2148-14-126 (PMC4113026; doi:10.1186/1471-2148-14-126)
Supplement: Additional file 6: Table S1 — Genera and species omitted in the analyses. [file 1471-2148-14-126-S6.docx]

**Table S1. Omitted genera and species in the analysis** (Although seven genera were omitted in the cladistic analysis, we present a temporary placement of these genera based on phylogenetic analysis and morphological traits (Fig. 17)).

| **Genus** | **Species** | **Geological Age** | **Reason for omission** |
| --- | --- | --- | --- |
| *Angarogramma* | *A. incertum* Ponomarenko, 1984 | Later Jurassic/Uda Fm. | Lack of exact description |
| *Apochrysogramma* | *P. bifaciatum* Yang et al., 2011 | Middle Jurassic/Jiulongshan Fm. | The wing partly preserved |
| *Kalligramma* | *K.* [*flexuosum*](http://lacewing.tamu.edu/Species-Catalogue/scdetailedresultsAdv.cfm?IDNum1=7011&%09%09%09%09%09IDquery1=Kalligramma&IDquery2=&IDquery3=flexuosum&IDquery4=&%09%09%09%09%09IDquery5=&IDquery6=&IDquery7=Panfilov&%09%09%09%09%09IDquery8=1968.??.??&IDquery9=&IDquery10=beginswith&IDquery11=Neuroptera&IDquery12=beginswith&IDquery14=Kalligrammatidae&IDquery15=beginswith&IDquery16=&IDquery17=beginswith&IDquery18=&IDquery19=beginswith&IDquery20=&IDquery21=beginswith&IDquery22=&IDquery23=beginswith&IDquery24=&IDquery25=beginswith&IDquery26=&IDquery27=beginswith&IDquery28=&IDquery29=beginswith&IDquery30=&IDquery31=beginswith&IDquery32=&IDquery33=beginswith&IDquery34=&IDquery35=beginswith&IDquery36=&IDquery37=beginswith&IDquery38=&IDquery39=beginswith&IDquery40=&IDquery41=beginswith&IDquery42=&IDquery43=beginswith&IDquery44=&IDquery45=beginswith&IDquery46=&IDquery47=beginswith&IDquery48=&IDquery49=beginswith&IDquery50=&IDquery51=is&IDquery52=&IDquery53=contains&IDquery54=&IDquery55=&IDquery56=&IDquery57=&IDquery58=&IDquery59=availableorunavailable&IDquery60=extantorfossil&IDquery13=0&IDquery61=Neuroptera&IDquery62=Kalligrammatidae&IDquery63=6053&IDquery64=&IDquery65=1) Panfilov, 1968 | Later Jurassic/Karabastau Fm. | Only hindwing preserved |
|  | *K.* [*haeckeli*](http://lacewing.tamu.edu/Species-Catalogue/scdetailedresultsAdv.cfm?IDNum1=6837&%09%09%09%09%09IDquery1=Kalligramma&IDquery2=&IDquery3=haeckeli&IDquery4=&%09%09%09%09%09IDquery5=&IDquery6=&IDquery7=Walther&%09%09%09%09%09IDquery8=1904.??.??&IDquery9=&IDquery10=beginswith&IDquery11=Neuroptera&IDquery12=beginswith&IDquery14=Kalligrammatidae&IDquery15=beginswith&IDquery16=&IDquery17=beginswith&IDquery18=&IDquery19=beginswith&IDquery20=&IDquery21=beginswith&IDquery22=&IDquery23=beginswith&IDquery24=&IDquery25=beginswith&IDquery26=&IDquery27=beginswith&IDquery28=&IDquery29=beginswith&IDquery30=&IDquery31=beginswith&IDquery32=&IDquery33=beginswith&IDquery34=&IDquery35=beginswith&IDquery36=&IDquery37=beginswith&IDquery38=&IDquery39=beginswith&IDquery40=&IDquery41=beginswith&IDquery42=&IDquery43=beginswith&IDquery44=&IDquery45=beginswith&IDquery46=&IDquery47=beginswith&IDquery48=&IDquery49=beginswith&IDquery50=&IDquery51=is&IDquery52=&IDquery53=contains&IDquery54=&IDquery55=&IDquery56=&IDquery57=&IDquery58=&IDquery59=availableorunavailable&IDquery60=extantorfossil&IDquery13=0&IDquery61=Neuroptera&IDquery62=Kalligrammatidae&IDquery63=6051&IDquery64=&IDquery65=1) Walther, 19the 04 | Later Jurassic/Solnhofen Fm. | Lack of exact description |
|  | *K.* [*jurarchegonium*](http://lacewing.tamu.edu/Species-Catalogue/scdetailedresultsAdv.cfm?IDNum1=9960&%09%09%09%09%09IDquery1=Kalligramma&IDquery2=&IDquery3=jurarchegonium&IDquery4=&%09%09%09%09%09IDquery5=&IDquery6=&IDquery7=J.$Zhang$*$H.$Zhang&%09%09%09%09%09IDquery8=2003.??.??&IDquery9=&IDquery10=beginswith&IDquery11=Neuroptera&IDquery12=beginswith&IDquery14=Kalligrammatidae&IDquery15=beginswith&IDquery16=&IDquery17=beginswith&IDquery18=&IDquery19=beginswith&IDquery20=&IDquery21=beginswith&IDquery22=&IDquery23=beginswith&IDquery24=&IDquery25=beginswith&IDquery26=&IDquery27=beginswith&IDquery28=&IDquery29=beginswith&IDquery30=&IDquery31=beginswith&IDquery32=&IDquery33=beginswith&IDquery34=&IDquery35=beginswith&IDquery36=&IDquery37=beginswith&IDquery38=&IDquery39=beginswith&IDquery40=&IDquery41=beginswith&IDquery42=&IDquery43=beginswith&IDquery44=&IDquery45=beginswith&IDquery46=&IDquery47=beginswith&IDquery48=&IDquery49=beginswith&IDquery50=&IDquery51=is&IDquery52=&IDquery53=contains&IDquery54=&IDquery55=&IDquery56=&IDquery57=&IDquery58=&IDquery59=availableorunavailable&IDquery60=extantorfossil&IDquery13=0&IDquery61=Neuroptera&IDquery62=Kalligrammatidae&IDquery63=15608&IDquery64=&IDquery65=1) J. Zhang & H. Zhang, 2003 | Middle Jurassic/Haifanggou Fm. | Only hindwing preserved |
|  | *K.* [*roycrowsoni*](http://lacewing.tamu.edu/Species-Catalogue/scdetailedresultsAdv.cfm?IDNum1=9881&%09%09%09%09%09IDquery1=Kalligramma&IDquery2=&IDquery3=roycrowsoni&IDquery4=&%09%09%09%09%09IDquery5=&IDquery6=&IDquery7=Jarzembowski&%09%09%09%09%09IDquery8=2001.??.??&IDquery9=&IDquery10=beginswith&IDquery11=Neuroptera&IDquery12=beginswith&IDquery14=Kalligrammatidae&IDquery15=beginswith&IDquery16=&IDquery17=beginswith&IDquery18=&IDquery19=beginswith&IDquery20=&IDquery21=beginswith&IDquery22=&IDquery23=beginswith&IDquery24=&IDquery25=beginswith&IDquery26=&IDquery27=beginswith&IDquery28=&IDquery29=beginswith&IDquery30=&IDquery31=beginswith&IDquery32=&IDquery33=beginswith&IDquery34=&IDquery35=beginswith&IDquery36=&IDquery37=beginswith&IDquery38=&IDquery39=beginswith&IDquery40=&IDquery41=beginswith&IDquery42=&IDquery43=beginswith&IDquery44=&IDquery45=beginswith&IDquery46=&IDquery47=beginswith&IDquery48=&IDquery49=beginswith&IDquery50=&IDquery51=is&IDquery52=&IDquery53=contains&IDquery54=&IDquery55=&IDquery56=&IDquery57=&IDquery58=&IDquery59=availableorunavailable&IDquery60=extantorfossil&IDquery13=0&IDquery61=Neuroptera&IDquery62=Kalligrammatidae&IDquery63=15192&IDquery64=&IDquery65=1) Jarzembowski, 2001 | Early Cretaceous/Wadhurst Clay Fm. | Lack of exact description |
|  | *K.* [*sharovi*](http://lacewing.tamu.edu/Species-Catalogue/scdetailedresultsAdv.cfm?IDNum1=7012&%09%09%09%09%09IDquery1=Kalligramma&IDquery2=&IDquery3=sharovi&IDquery4=&%09%09%09%09%09IDquery5=&IDquery6=&IDquery7=Panfilov&%09%09%09%09%09IDquery8=1968.??.??&IDquery9=&IDquery10=beginswith&IDquery11=Neuroptera&IDquery12=beginswith&IDquery14=Kalligrammatidae&IDquery15=beginswith&IDquery16=&IDquery17=beginswith&IDquery18=&IDquery19=beginswith&IDquery20=&IDquery21=beginswith&IDquery22=&IDquery23=beginswith&IDquery24=&IDquery25=beginswith&IDquery26=&IDquery27=beginswith&IDquery28=&IDquery29=beginswith&IDquery30=&IDquery31=beginswith&IDquery32=&IDquery33=beginswith&IDquery34=&IDquery35=beginswith&IDquery36=&IDquery37=beginswith&IDquery38=&IDquery39=beginswith&IDquery40=&IDquery41=beginswith&IDquery42=&IDquery43=beginswith&IDquery44=&IDquery45=beginswith&IDquery46=&IDquery47=beginswith&IDquery48=&IDquery49=beginswith&IDquery50=&IDquery51=is&IDquery52=&IDquery53=contains&IDquery54=&IDquery55=&IDquery56=&IDquery57=&IDquery58=&IDquery59=availableorunavailable&IDquery60=extantorfossil&IDquery13=0&IDquery61=Neuroptera&IDquery62=Kalligrammatidae&IDquery63=6054&IDquery64=&IDquery65=1) Panfilov, 1968 | Later Jurassic/Karabastau Fm. | Only hindwing preserved |
| *Kalligrammina* | *K.* [*areolata*](http://lacewing.tamu.edu/Species-Catalogue/scdetailedresultsAdv.cfm?IDNum1=7053&%09%09%09%09%09IDquery1=Kalligrammina&IDquery2=&IDquery3=areolata&IDquery4=&%09%09%09%09%09IDquery5=&IDquery6=&IDquery7=Panfilov$in$Dolin$et$al.&%09%09%09%09%09IDquery8=1980.01.27&IDquery9=&IDquery10=beginswith&IDquery11=Neuroptera&IDquery12=beginswith&IDquery14=Kalligrammatidae&IDquery15=beginswith&IDquery16=&IDquery17=beginswith&IDquery18=&IDquery19=beginswith&IDquery20=&IDquery21=beginswith&IDquery22=&IDquery23=beginswith&IDquery24=&IDquery25=beginswith&IDquery26=&IDquery27=beginswith&IDquery28=&IDquery29=beginswith&IDquery30=&IDquery31=beginswith&IDquery32=&IDquery33=beginswith&IDquery34=&IDquery35=beginswith&IDquery36=&IDquery37=beginswith&IDquery38=&IDquery39=beginswith&IDquery40=&IDquery41=beginswith&IDquery42=&IDquery43=beginswith&IDquery44=&IDquery45=beginswith&IDquery46=&IDquery47=beginswith&IDquery48=&IDquery49=beginswith&IDquery50=&IDquery51=is&IDquery52=&IDquery53=contains&IDquery54=&IDquery55=&IDquery56=&IDquery57=&IDquery58=&IDquery59=availableorunavailable&IDquery60=extantorfossil&IDquery13=0&IDquery61=Neuroptera&IDquery62=Kalligrammatidae&IDquery63=6056&IDquery64=&IDquery65=1) Panfilov, 1980 | Later Jurassic/Karabastau Fm. | Only hindwing preserved |
| *Kalligrammula* | *K.* [*atra*](http://lacewing.tamu.edu/Species-Catalogue/scdetailedresultsAdv.cfm?IDNum1=9187&%09%09%09%09%09IDquery1=Kalligrammula&IDquery2=&IDquery3=atra&IDquery4=&%09%09%09%09%09IDquery5=&IDquery6=&IDquery7=Ponomarenko&%09%09%09%09%09IDquery8=1992.??.??&IDquery9=&IDquery10=beginswith&IDquery11=Neuroptera&IDquery12=beginswith&IDquery14=Kalligrammatidae&IDquery15=beginswith&IDquery16=&IDquery17=beginswith&IDquery18=&IDquery19=beginswith&IDquery20=&IDquery21=beginswith&IDquery22=&IDquery23=beginswith&IDquery24=&IDquery25=beginswith&IDquery26=&IDquery27=beginswith&IDquery28=&IDquery29=beginswith&IDquery30=&IDquery31=beginswith&IDquery32=&IDquery33=beginswith&IDquery34=&IDquery35=beginswith&IDquery36=&IDquery37=beginswith&IDquery38=&IDquery39=beginswith&IDquery40=&IDquery41=beginswith&IDquery42=&IDquery43=beginswith&IDquery44=&IDquery45=beginswith&IDquery46=&IDquery47=beginswith&IDquery48=&IDquery49=beginswith&IDquery50=&IDquery51=is&IDquery52=&IDquery53=contains&IDquery54=&IDquery55=&IDquery56=&IDquery57=&IDquery58=&IDquery59=availableorunavailable&IDquery60=extantorfossil&IDquery13=0&IDquery61=Neuroptera&IDquery62=Kalligrammatidae&IDquery63=6059&IDquery64=&IDquery65=1) Ponomarenko, 1992 | Early Cretaceous/Shine-Khuduk Fm. | Lack of exact description |
|  | *K. senckenbergiana* Handlirsch, 1919 | Later Jurassic/Solnhofen Fm. | Lack of exact description |
| *Kallihemerobius* | *K. aciedentatus* sp. nov. | Middle Jurassic/Jiulongshan Fm. | Only hindwing preserved |
|  | *K.* [*pleioneurus*](http://lacewing.tamu.edu/Species-Catalogue/scdetailedresultsAdv.cfm?IDNum1=9725&%09%09%09%09%09IDquery1=Kallihemerobius&IDquery2=&IDquery3=pleioneurus&IDquery4=&%09%09%09%09%09IDquery5=&IDquery6=&IDquery7=Ren$*$Oswald&%09%09%09%09%09IDquery8=2002.??.??&IDquery9=&IDquery10=beginswith&IDquery11=Neuroptera&IDquery12=beginswith&IDquery14=Kalligrammatidae&IDquery15=beginswith&IDquery16=&IDquery17=beginswith&IDquery18=&IDquery19=beginswith&IDquery20=&IDquery21=beginswith&IDquery22=&IDquery23=beginswith&IDquery24=&IDquery25=beginswith&IDquery26=&IDquery27=beginswith&IDquery28=&IDquery29=beginswith&IDquery30=&IDquery31=beginswith&IDquery32=&IDquery33=beginswith&IDquery34=&IDquery35=beginswith&IDquery36=&IDquery37=beginswith&IDquery38=&IDquery39=beginswith&IDquery40=&IDquery41=beginswith&IDquery42=&IDquery43=beginswith&IDquery44=&IDquery45=beginswith&IDquery46=&IDquery47=beginswith&IDquery48=&IDquery49=beginswith&IDquery50=&IDquery51=is&IDquery52=&IDquery53=contains&IDquery54=&IDquery55=&IDquery56=&IDquery57=&IDquery58=&IDquery59=availableorunavailable&IDquery60=extantorfossil&IDquery13=0&IDquery61=Neuroptera&IDquery62=Kalligrammatidae&IDquery63=14905&IDquery64=&IDquery65=1) Ren & Oswald, 2002 | Middle Jurassic/Jiulongshan Fm. | Only hindwing preserved |
| *Limnogramma* | *L. mira* Ren, 2003 | Early Cretaceous/Yixian Fm. | Only hindwing preserved |
| *Meioneurites* | *M.* [*schlosseri*](http://lacewing.tamu.edu/Species-Catalogue/scdetailedresultsAdv.cfm?IDNum1=7017&%09%09%09%09%09IDquery1=Meioneurites&IDquery2=&IDquery3=schlosseri&IDquery4=&%09%09%09%09%09IDquery5=&IDquery6=&IDquery7=Handlirsch&%09%09%09%09%09IDquery8=%5b1906.10.??%5d&IDquery9=&IDquery10=beginswith&IDquery11=Neuroptera&IDquery12=beginswith&IDquery14=Kalligrammatidae&IDquery15=beginswith&IDquery16=&IDquery17=beginswith&IDquery18=&IDquery19=beginswith&IDquery20=&IDquery21=beginswith&IDquery22=&IDquery23=beginswith&IDquery24=&IDquery25=beginswith&IDquery26=&IDquery27=beginswith&IDquery28=&IDquery29=beginswith&IDquery30=&IDquery31=beginswith&IDquery32=&IDquery33=beginswith&IDquery34=&IDquery35=beginswith&IDquery36=&IDquery37=beginswith&IDquery38=&IDquery39=beginswith&IDquery40=&IDquery41=beginswith&IDquery42=&IDquery43=beginswith&IDquery44=&IDquery45=beginswith&IDquery46=&IDquery47=beginswith&IDquery48=&IDquery49=beginswith&IDquery50=&IDquery51=is&IDquery52=&IDquery53=contains&IDquery54=&IDquery55=&IDquery56=&IDquery57=&IDquery58=&IDquery59=availableorunavailable&IDquery60=extantorfossil&IDquery13=0&IDquery61=Neuroptera&IDquery62=Kalligrammatidae&IDquery63=7192&IDquery64=&IDquery65=1) Handlirsch, 1906 | Later Jurassic/Solnhofen Fm. | Lack of exact description |
| *Palparites* | *P.* [*deichmuelleri*](http://lacewing.tamu.edu/Species-Catalogue/scdetailedresultsAdv.cfm?IDNum1=7060&%09%09%09%09%09IDquery1=Palparites&IDquery2=&IDquery3=deichmuelleri&IDquery4=&%09%09%09%09%09IDquery5=&IDquery6=&IDquery7=Handlirsch&%09%09%09%09%09IDquery8=%5b1906.10.??%5d&IDquery9=&IDquery10=beginswith&IDquery11=Neuroptera&IDquery12=beginswith&IDquery14=Kalligrammatidae&IDquery15=beginswith&IDquery16=&IDquery17=beginswith&IDquery18=&IDquery19=beginswith&IDquery20=&IDquery21=beginswith&IDquery22=&IDquery23=beginswith&IDquery24=&IDquery25=beginswith&IDquery26=&IDquery27=beginswith&IDquery28=&IDquery29=beginswith&IDquery30=&IDquery31=beginswith&IDquery32=&IDquery33=beginswith&IDquery34=&IDquery35=beginswith&IDquery36=&IDquery37=beginswith&IDquery38=&IDquery39=beginswith&IDquery40=&IDquery41=beginswith&IDquery42=&IDquery43=beginswith&IDquery44=&IDquery45=beginswith&IDquery46=&IDquery47=beginswith&IDquery48=&IDquery49=beginswith&IDquery50=&IDquery51=is&IDquery52=&IDquery53=contains&IDquery54=&IDquery55=&IDquery56=&IDquery57=&IDquery58=&IDquery59=availableorunavailable&IDquery60=extantorfossil&IDquery13=0&IDquery61=Neuroptera&IDquery62=Kalligrammatidae&IDquery63=9455&IDquery64=&IDquery65=1) Handlirsch, 1906 | Later Jurassic/Solnhofen Fm. | Lack of exact description |
| *Protokalligramma* | *P. bifasciatum* Yang et al., 2011 | Middle Jurassic/Jiulongshan Fm. | The wing partly preserved |
| *Sinokalligramma* | *S. jurassicum* Zhang, 2003 | Middle Jurassic/Daohugou Fm. | Only hindwing preserved |
