# Supplementary material for: Mesozoic lacewings from China provide phylogenetic insight into evolution of the Kalligrammatidae (Neuroptera)
Source: BMC Evol Biol. 2014 Jun 9;14:126. doi: 10.1186/1471-2148-14-126 (PMC4113026; doi:10.1186/1471-2148-14-126)
Supplement: Additional file 7: Table S2 — Taxon–character-state matrix. [file 1471-2148-14-126-S7.docx]

**Table 2. Taxon–character-state data matrix**

| ***Taxa*** | **01** | **02** | **03** | **04** | **05** | **06** | **07** | **08** | **09** | **10** | **11** | **12** | **13** | **14** | **15** | **16** | **17** | **18** | **19** | **20** | **21** | **22** | **23** | **24** | **25** | **26** | **27** | **28** | **29** | **30** |
| --- | --- | --- | --- | --- | --- | --- | --- | --- | --- | --- | --- | --- | --- | --- | --- | --- | --- | --- | --- | --- | --- | --- | --- | --- | --- | --- | --- | --- | --- | --- |
| *Saucrosmylus sambneurus* | 0 | 0 | 2 | 1 | 1 | 0 | 0 | 0 | 0 | 1 | 0 | 0 | 0 | 0 | 0 | 0 | 0 | 0 | 0 | 0 | 0 | 3 | 0 | 2 | 0 | 0 | 0 | 0 | 0 | 0 |
| *Panfilovia acuminatus* | 0 | 0 | 2 | 1 | 1 | 0 | 1 | 0 | 0 | 1 | 0 | 0 | 0 | 1 | 0 | 0 | 0 | 0 | 0 | 0 | 0 | 3 | 1 | 2 | 0 | 0 | 0 | 0 | 0 | 0 |
| *Aetheogramma speciosa* | 0 | 0 | 0 | 0 | 0 | 1 | 1 | 1 | 0 | 0 | 0 | 0 | 0 | 0 | 1 | 0 | 0 | 1 | 0 | 0 | 0 | 0 | 1 | 2 | 0 | 0 | 0 | 0 | 0 | 0 |
| *Grammolingia boi* | 0 | 0 | 1 | 0 | 0 | 1 | 1 | 0 | 0 | 0 | 0 | 0 | 0 | 0 | 0 | 0 | 0 | 0 | 0 | 0 | 0 | 3 | 0 | 0 | 0 | 0 | 0 | 0 | 0 | 0 |
| *Abrigramma calophleba* | 0 | 1 | 2 | 0 | ? | ? | 1 | 0 | 0 | 1 | 0 | 0 | 0 | 1 | 1 | 0 | 0 | 1 | 0 | 1 | 1 | 0 | 0 | 2 | 0 | 2 | ? | ? | 0 | 1 |
| *Affingramma myrioneura* | 0 | 2 | 2 | 2 | 1 | 1 | 1 | 0 | 1 | 0 | 1 | 0 | 1 | 3 | 1 | 0 | 0 | 0 | 0 | 0 | 0 | 0 | 3 | 3 | 0 | 2 | 2 | 1 | 0 | 2 |
| *Huiyingogramma formosum* | 1 | 0 | 2 | 1 | 1 | ? | 1 | 0 | 0 | 0 | 1 | 0 | 0 | 2 | 0 | 0 | 0 | 0 | 0 | 0 | 0 | 2 | 3 | 4 | 0 | 2 | 2 | ? | ? | ? |
| *Ithigramma multinervia* | ? | 1 | 0 | 0 | 1 | 1 | 1 | 0 | 0 | ? | 0 | 0 | 0 | ? | 1 | 0 | 0 | ? | 0 | 0 | 1 | ? | ? | ? | 1 | 1 | 1 | ? | 0 | 3 |
| *Kallihemerobius aciedentatus* | 0 | 2 | 2 | 2 | 1 | ? | 1 | 0 | 2 | 0 | 1 | 0 | 2 | ? | 1 | 0 | 0 | 0 | 0 | 0 | 0 | 2 | ? | ? | 0 | ? | 3 | ? | 0 | 2 |
| *Kallihemerobius feroculus* | 1 | 2 | 2 | 2 | 1 | ? | 1 | 0 | 2 | 0 | 1 | 0 | 2 | 3 | 1 | 0 | 0 | 0 | 0 | 0 | 0 | 2 | 2 | ? | 0 | ? | 3 | ? | 0 | 2 |
| *Kalligramma brachyrhncha* | 0 | 0 | 2 | 1 | 1 | 1 | 1 | 0 | 0 | 1 | 0 | 0 | 0 | 1 | 1 | 0 | 0 | 0 | 0 | 0 | 0 | 1 | 1 | 2 | 0 | 2 | 2 | 1 | 0 | 2 |
| *Kalligramma circularia* | 0 | 0 | 1 | 1 | 1 | 1 | 1 | 0 | 0 | 1 | 0 | 0 | 0 | ? | 1 | 0 | 0 | 0 | 0 | 0 | 0 | 1 | ? | ? | 0 | 2 | 2 | 1 | 0 | 2 |
| *Kalligramma liaoningense* | 0 | 0 | 2 | 1 | 1 | 1 | 1 | 0 | 0 | 1 | 0 | 0 | 0 | 1 | 1 | 0 | 0 | 0 | 0 | 0 | 0 | 1 | 1 | 2 | 0 | 2 | 2 | 1 | 0 | ? |
| *Kalligramma multinerve* | 0 | 0 | 2 | 1 | ? | 1 | 1 | 0 | 0 | 1 | 0 | 1 | 0 | 1 | 1 | 0 | ? | 0 | 0 | 0 | 0 | ? | 1 | 2 | 0 | 1 | 2 | ? | ? | ? |
| *Kalligramma turutanovae* | 0 | 0 | 2 | 1 | 1 | ? | 1 | 0 | 0 | 1 | 0 | 0 | 0 | 1 | 1 | 0 | 1 | 0 | 0 | 0 | 0 | 1 | ? | ? | 0 | 2 | 2 | ? | ? | ? |
| *Kalligrammula karatavica* | ? | 0 | ? | ? | 1 | 1 | 1 | 0 | 0 | 0 | 1 | 0 | 0 | 1 | 1 | 0 | 0 | 0 | 0 | 0 | 0 | 4 | 3 | ? | 0 | 2 | ? | ? | ? | ? |
| *Lithogramma oculatum* | ? | 2 | 2 | 1 | 1 | ? | 1 | 0 | 0 | 0 | 1 | 0 | 0 | 3 | 1 | 0 | 0 | 0 | 0 | 0 | 0 | 2 | ? | ? | 0 | 1 | 2 | ? | ? | ? |
| *Meioneurites spectabilis* | ? | 0 | 0 | ? | ? | ? | 1 | 0 | 0 | ? | ? | 0 | 0 | ? | 1 | 1 | ? | 0 | 0 | 0 | 0 | ? | ? | ? | 0 | 2 | 1 | 1 | 0 | 1 |
| *Meioneurites villosus* | ? | 0 | 0 | ? | ? | ? | 1 | 0 | 0 | 0 | 0 | 0 | 0 | 2 | 1 | 1 | 0 | 0 | 0 | 0 | 0 | 0 | 0 | 0 | 0 | 2 | ? | 1 | 0 | ? |
| *Oregramma aureolusa* | 0 | 1 | 0 | 0 | 1 | ? | 1 | 0 | 0 | 1 | 0 | 0 | 0 | 2 | 1 | 0 | 0 | 1 | 0 | 0 | 1 | 0 | 0 | 2 | 0 | 1 | 0 | ? | 0 | 2 |
| *Oregramma gloriosa* | 0 | 1 | 0 | 0 | 1 | 1 | 1 | 0 | 0 | 1 | 0 | 0 | 0 | 2 | 1 | 0 | 0 | 1 | 0 | 0 | 1 | 0 | 0 | 1 | 0 | 1 | 3 | 2 | 0 | ? |
| *Oregramma illecebrosa* | 0 | 1 | 0 | 0 | 1 | 1 | 1 | 0 | 0 | 1 | 0 | 0 | 0 | 2 | 1 | 0 | 0 | 1 | 0 | 0 | 1 | 0 | 0 | 1 | 0 | 1 | 3 | 2 | 1 | ? |
| *Stelligramma allochroma* | 1 | 0 | 2 | 1 | ? | ? | 1 | 0 | 0 | ? | 1 | 1 | 0 | 1 | 1 | 0 | 0 | 0 | 0 | 0 | 0 | 2 | 3 | 3 | 0 | ? | ? | ? | 0 | 2 |
| *Sophogramma eucallum* | 1 | 0 | 2 | 1 | 1 | 1 | 1 | 0 | 0 | 1 | 1 | 1 | 0 | 2 | 0 | 0 | 0 | 0 | 1 | 0 | 0 | 2 | 0 | 4 | 0 | 2 | 0 | 1 | 0 | 0 |
| *Sophogramma lii* | 1 | 0 | 2 | 1 | 1 | 1 | 1 | 0 | 0 | 1 | 1 | 1 | 0 | 2 | 0 | 0 | 0 | 0 | 1 | 0 | 0 | 2 | 0 | 4 | 0 | 2 | 0 | 1 | 0 | 0 |
| *Sophogramma papilionacea* | 1 | 0 | 2 | 1 | ? | ? | 1 | 0 | 0 | 1 | 1 | 1 | 0 | 2 | 0 | 0 | 0 | 0 | 1 | 0 | 0 | 2 | 0 | 4 | 0 | 2 | 0 | 1 | 0 | 0 |
| *Sophogramma plecophlebia* | 1 | 0 | 2 | 1 | 1 | 1 | 1 | 0 | 0 | 1 | 1 | 1 | 0 | 2 | 0 | 0 | 0 | 0 | 1 | 0 | 0 | 2 | 0 | 4 | 0 | 2 | 0 | 1 | 0 | 0 |
| *Sophogramma pingquanica* | 1 | 0 | 2 | 1 | 1 | ? | 1 | 0 | 0 | 1 | 1 | 1 | 0 | 2 | 0 | 0 | 0 | 0 | 1 | 0 | 0 | 2 | 0 | 4 | 0 | 2 | 0 | 1 | 0 | 0 |
